# Spatial Layouts of Low‐Entropy Hydration Shells Guide Protein Binding

**DOI:** 10.1002/gch2.202300022

**Published:** 2023-05-02

**Authors:** Lin Yang, Shuai Guo, Chenchen Liao, Chengyu Hou, Shenda Jiang, Jiacheng Li, Xiaoliang Ma, Liping Shi, Lin Ye, Xiaodong He

**Affiliations:** ^1^ National Key Laboratory of Science and Technology on Advanced Composites in Special Environments Center for Composite Materials and Structures Harbin Institute of Technology Harbin 150080 P. R. China; ^2^ School of Aerospace Mechanical and Mechatronic Engineering The University of Sydney NSW 2006 Australia; ^3^ School of Electronics and Information Engineering Harbin Institute of Technology Harbin 150080 P. R. China; ^4^ School of System Design and Intelligent Manufacturing Southern University of Science and Technology Shenzhen 518055 P. R. China; ^5^ Shenzhen STRONG Advanced Materials Research Institute Co., Ltd Shenzhen 518035 P. R. China

**Keywords:** binding site, Gibbs free energy, hydration shell, low entropy, protein–protein interactions

## Abstract

Protein–protein binding enables orderly biological self‐organization and is therefore considered a miracle of nature. Protein‒protein binding is driven by electrostatic forces, hydrogen bonding, van der Waals force, and hydrophobic interactions. Among these physical forces, only hydrophobic interactions can be considered long‐range intermolecular attractions between proteins due to the electrostatic shielding of surrounding water molecules. Low‐entropy hydration shells around proteins drive hydrophobic attraction among them that essentially coordinate protein‒protein binding. Here, an innovative method is developed for identifying low‐entropy regions of hydration shells of proteins by screening off pseudohydrophilic groups on protein surfaces and revealing that large low‐entropy regions of the hydration shells typically cover the binding sites of individual proteins. According to an analysis of determined protein complex structures, shape matching between a large low‐entropy hydration shell region of a protein and that of its partner at the binding sites is revealed as a universal law. Protein‒protein binding is thus found to be mainly guided by hydrophobic collapse between the shape‐matched low‐entropy hydration shells that is verified by bioinformatics analyses of hundreds of structures of protein complexes, which cover four test systems. A simple algorithm is proposed to accurately predict protein binding sites.

## Graphical abstract

An innovative method is developed for identifying low‐entropy regions of hydration shells of proteins by screening off pseudohydrophilic groups. Large low‐entropy regions of the hydration shells typically cover the binding sites of proteins. Shape matching between a large low‐entropy hydration shell region of a protein and that of its partner at the binding sites is revealed as a universal law.

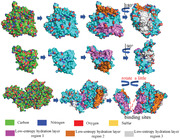

## 1 Introduction

Proteins serve a variety of important functions in organisms. A protein's intrinsic biological functions are normally expressed via precise binding with another protein (i.e., a ligand), involving in nearly all physiological processes, including immune response, DNA replication, enzyme inhibition, and the signal transduction. Protein‒protein binding is a spontaneous physical contact of high specificity, established between two specific protein molecules, and protein‒protein binding is highly rare in extracellular fluid.^[^ ^1^ ^]^ At present, it is generally accepted that protein‒protein binding does not violate the second law of thermodynamics because the binding state is most likely a state of maximum entropy of the extracellular or intracellular aqueous solution system. The physical mechanism responsible for protein‒protein binding can be considered the most important mechanism of bioogical self‐organization, functionalization, and diversity.

The July 1, 2005 issue of Science published a list of 125 important questions in science, one of the questions is “How do proteins find their partners?”.^[^ ^2^ ^]^ Even though significant progress has been made toward the structural determination of protein complexes, many complexes that are crucial drug targets for cancer and diseases remain difficult to determine experimentally.^[^ ^3^, ^4^ ^]^ Computational prediction of the peptide‐protein and protein‐protein binding using machine learning methods is advancing, speeding up immunogenic peptide screening and facilitate protein‐based drug design.^[^ ^5^, ^6^, ^7^ ^]^ Experimental approaches like double‐mutant cycles and paramagnetic relaxation enhancement have been used to investigate protein–protein binding mechanisms, providing information about potential transition states and intermediates, but these data's are often indirect or limited to the specific protein.^[^ ^8^, ^9^ ^]^ Moreover, it is difficult to obtain the desired protein binding results by traditional molecular dynamics simulation.^[^ ^10^ ^]^ It remains a challenge to determine a common and direct biophysical mechanism of protein–protein interactions. Protein‒protein binding is one of the miracles of nature that human technology finds quite difficult to follow due to numberless possibilities in theory of the rotational conformation potentially sampled by two or more protein molecules as they interact.^[^ ^11^ ^]^ Protein‒protein docking focuses on predicting the natural complex structure of protein‒protein binding in computational informatics.^[^ ^2^, ^11^ ^]^ Research on protein‒protein docking has become more popular due to its potential to predict important protein‒protein interactions (PPIs), such as the targeting action of recombinant protein drugs and antibody drugs,^[^ ^12^, ^13^ ^]^ a variety of conformational search strategies have been applied to predict protein docking.^[^ ^14^, ^15^ ^]^ Although searching algorithms normally aim to achieve the optimized conformations for protein complexes with the minimized free energy of the whole system, sampling of the conformational space in protein‒protein docking is still challenging due to the lack of understanding of the entropy state of the hydration shells of individual proteins.^[^ ^16^, ^17^ ^]^

Protein‒protein binding was mainly governed by electrostatic forces, hydrogen bonding, van der Waals forces, and hydrophobic interactions. However, among these physical forces, only hydrophobic interactions can be viewed as long‐range intermolecular attractions between protein molecules in aqueous solutions due to the electrostatic shielding of water molecules between proteins. Water molecules are obviously more electrically polarized than the hydrophilic groups of proteins ^[^ ^18^ ^]^ (see Figure S1, Supporting Information). Protein surface hydrophilic groups tend to hydrogen bond with surrounding strong polar water molecules in the hydration shell, thereby preventing the surface hydrogen bond donors of a protein from randomly hydrogen‐bonding with the hydrogen bond acceptors of another protein, and vice versa, namely, preventing erroneous protein‒protein binding in unsaturated aqueous solution.^[^ ^19^, ^20^, ^21^, ^22^, ^23^, ^24^ ^]^ Otherwise, it cannot be explained why erroneous protein‒protein binding is highly rare in water solution and very common in nonaqueous solution.^[^ ^25^ ^]^

The hydrophobic effect has been considered essential to protein‒protein binding, and is governed by a balance between enthalpy and entropy contributions from the hydration shells and proteins.^[^ ^26^, ^27^ ^]^ An accurate description of solvation free energies without implicit solvent models is a challenging task in computer simulations.^[^ ^28^, ^29^ ^]^ Recent progress in molecular dynamics simulations and NMR spectroscopy has led to new insights into fluctuations of water structure at hydrated biointerfaces and the processes of vibrational relaxation.^[^ ^18^, ^30^, ^36^ ^]^ However, it remains unclear how water dynamics affect the protein‒protein binding process.

**Figure 1:**
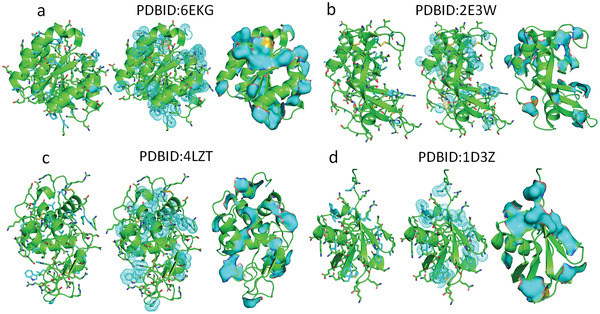
Illustrations of cyan hydrophobic side‐chains protruding on protein surfaces a) Chemotaxis protein CheY, b) Ribonuclease A, c) Hen egg‐white lysozyme (HEWL), d) Ubiquitin. Correspond to PDB structures: 6EKG, 2E3W, 4LZT, and 1D3Z.

The structure and function of proteins are strongly influenced by their hydration shells.^[^ ^31^ ^]^ Hydrophobic side chains protruding on protein surfaces are very common in experimentally determined protein structures (see **Figure** **1**). The hydration shell (i.e., Hydration layer) around a hydrophobic group of a protein has been experimentally found to have dynamics distinct from the bulk water to a distance of 1–5 nm.^[^ ^19^, ^37^, ^38^ ^]^ Water molecules slow down greatly when they enter the hydration shell of the protein, and their speed is reduced by 2–5 times.^[^ ^19^, ^39^ ^]^ Experimental evidence has shown that the hydrogen bonding network of the water molecules within protein hydration layer are much more ordered than those of the bulk; therefore, their rotation and number of possible configurations are heavily reduced,^[^ ^26^, ^40^ ^]^ resulting in lower entropy levels within hydration shell than bulk water molecules.^[^ ^19^, ^37^, ^41^, ^42^ ^]^ Thus, the protein‒protein binding phenomenon should start from the long‐range hydrophobic attraction between low‐entropy regions of the protein's hydration shells.^[^ ^43^, ^44^ ^]^ Jayangika and Ryan et al have reported that hydration water dynamics were highly heterogeneous and hydrophobic protein surfaces favored a more tetrahedral solvation layer and less entropy.^[^ ^26^, ^45^ ^]^ However, practical methods for identifying low‐entropy regions of protein hydration shells have not yet been developed. In this study, we describe an innovative method for identifying low‐entropy hydration shells of proteins by screening off pseudohydrophilic groups on their surfaces.

## 2 Results and Discussion

### 2.1 Intramolecular Hydrogen Bonds Formed by Protein Folding

Intramolecular hydrogen (H)‐bonds play an extremely important role in stabilizing protein structures. To form these intramolecular hydrogen bonds, nascent unfolded polypeptide chains need to escape from hydrogen bonding with surrounding polar water molecules under solution conditions that require entropy‐enthalpy compensation, according to the Gibbs free energy equation and the change in enthalpy.^[^ ^46^ ^]^ Therefore, the folding process of a protein is accompanied by the loss of hydrophilicity of many hydrophilic groups of the polypeptide due to the formation of intramolecular hydrogen bonds. This means that protein folding always makes some hydrophilic groups of polypeptide chains free from hydrogen bonding with surrounding water molecules. This should be attributed to the entropy‐enthalpy compensation during the folding of the secondary and tertiary structures. Protein folding indeed makes many hydrophilic groups lose their hydrophilicity, namely, the hydrophilic groups lose their ability to form hydrogen bonds with surrounding water molecules. Thus, there are reasons to doubt some hydrophilic groups on protein surfaces that do not express their hydrophilicity. It should be emphasized that the distributions of hydrophobic and hydrophilic groups on the surface of a protein are the result of protein folding. The protein surface hydration dynamics are determined not only by the distribution of hydrophilic and hydrophobic groups on a protein surface but also by interactions among these surface hydrophilic and hydrophobic groups.^[^ ^31^, ^47^ ^]^ Native structures of proteins are normally characterized by a cluttered distribution of hydrophilic and hydrophobic groups on the surfaces, and the interactions among these neighboring hydrophilic and hydrophobic groups most likely stop some hydrophilic groups from playing the roles of hydrogen bond acceptors or hydrogen bond donors with surrounding water molecules. Recent experimental evidence has shown that protein surface hydration dynamics are highly heterogeneous over the global protein surface.^[^ ^31^, ^45^, ^47^ ^]^ This indicates the existence of large low‐entropy regions of protein hydration shells and pseudohydrophilic groups on protein surfaces.

### 2.2 Pseudohydrophilic Groups on Protein Surfaces

**Figure 2:**
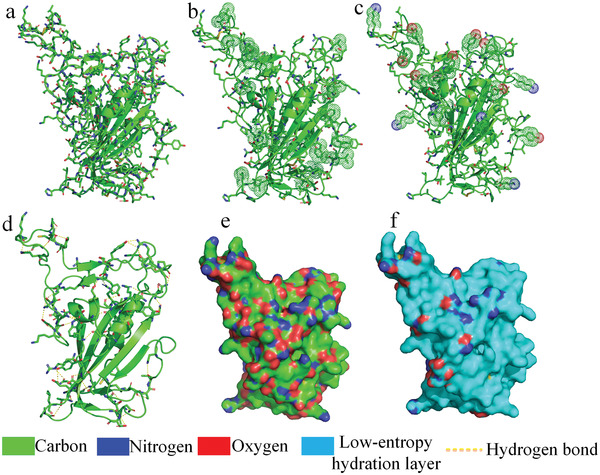
a) Molecular structure of the RBD of the spike protein of SARS‐CoV‐2. b) Intramolecular hydrogen bonded groups on the surface of the protein (hydrogen bonds are highlighted by yellow dashed lines). c) Hydrophobic side chains protruding on the surface of the protein (highlighted by dots). d) The side chains of tryptophan, tyrosine, and lysine protruding on the surface of the protein (highlighted by dots). e) Distribution of hydrophilic nitrogen and oxygen atoms on the protein surface. f) Distribution of the low‐entropy hydration layer on the surface of the protein.

There are at least three reasons that can cause protein surface hydrophilic groups to not express hydrophilicity. First, it is important to note that proteins normally have an abundance of intramolecular hydrogen bonds. For example, protein secondary structures arise from the hydrogen bonds formed between the amide proton and the carbonyl oxygen of the polypeptide backbone. Intramolecular hydrogen bonds play an extremely important role in stabilizing protein structures. To form these intramolecular hydrogen bonds, nascent unfolded polypeptide chains need to escape from hydrogen bonding with surrounding polar water molecules that require entropy‐enthalpy compensation during protein folding, according to the Gibbs free energy equation. It is worth noting that some residues are distant in sequence but form intramolecular hydrogen bonds with each other in the protein native structure. These hydrogen bonds are also stabilized by entropy‐enthalpy compensation during folding of the tertiary structures. This means that protein surface intramolecular hydrogen bonds saturate many of the hydrogen bonds formed by surface hydrophilic groups of proteins. The receptor‐binding domain (RBD) of the spike protein of the Omicron BA.4/5 variant of severe acute respiratory syndrome coronavirus 2 (SARS‐CoV‐2) is used to illustrate the intramolecular hydrogen bonds formed by surface hydrophilic groups of proteins (see **Figure** **2**a and b) ^[^ ^48^ ^]^ . Therefore, we can consider that these intramolecular hydrogen‐bonded surface hydrophilic groups cannot destroy the surrounding hydrophobic group‐induced low‐entropy water molecule network because their hydrophilicity is expressed by intramolecular hydrogen bonds. On protein surfaces, the hydration shells covering these intramolecular hydrogen‐bonded hydrophilic groups can be considered low‐entropy hydration shells, namely, these hydrophilic groups are pseudohydrophilic.

Second, it is worth noting that protein folding normally cannot orient all the hydrophobic side chains of a protein inward to join the hydrophobic core of the protein structure. Almost all experimentally determined proteins have hydrophobic side chains located on their surfaces.^[^ ^49^, ^50^, ^51^, ^52^ ^]^ In experimentally determined protein structures, there are always some backbone carbonyl oxygen atoms and amide hydrogen atoms exposed at the protein surfaces. Some surface hydrophobic side chains protrude outward to surrounding water molecules that most likely shield the hydrophilic backbone carbonyl oxygen atoms and amide hydrogen atoms from water molecules. For example, according to all hydrophobicity scales, isoleucine, valine, leucine, and phenylalanine residues are highly hydrophobic, even if the residues contain hydrophilic backbone carbonyl oxygen atoms and amide hydrogen atoms. Hydrophobic side‐chains of isoleucine, valine, leucine, and phenylalanine residues on protein surfaces expel surrounding water molecules to van der Waals radius (0.3 to 0.6 nm) to form the low‐entropy hydration shells (i.e., ordered water molecule cages). Van der Waals radius is much larger than the hydrogen bonding distance (0.3 nm). In this way, the ordered water molecules in the low‐entropy hydration shells around hydrophobic side chains are inhibited from fluctuating and rearrangement and are therefore prevented from frequently hydrogen bonding with the backbone carbonyl oxygen atoms and amide hydrogen atoms. This means that the highly hydrophobic side chains can shield the hydrophilicity of the backbone atoms to a certain extent. Thus, the hydration shells covering the backbone carbonyl oxygen atoms and amide hydrogen atoms of these hydrophobic residues can be regarded as low‐entropy hydration shells because the hydrogen‐bond rearrangements between the backbone hydrophilic atoms and surrounding water molecules are inhibited. The RBD protein is also used to illustrate the shielding effect of the protruding hydrophobic side chains (see Figure 2c). It is worth noting that the side chains of tyrosine, tryptophan, cysteine, methionine, lysine, etc., residues also contain highly hydrophobic structures (i.e., alkyl and benzene rings) that can shield their main‐chain structures. The hydration shells surrounding the backbone carbonyl oxygen groups and the amide hydrogen groups of these residues should also be considered low‐entropy hydration shells (see Figure 2c); namely, we can consider that these backbone carbonyl oxygen groups and the amide hydrogen groups are also pseudohydrophilic. It is worth noting that glycine has very weak hydrophilicity.^[^ ^53^, ^54^, ^55^ ^]^ The hydration shells surrounding the backbone carbonyl oxygen groups and the amide hydrogen groups of the residue should be considered low‐entropy hydration shells.

Third, according to the different hydrophobicity scales of amino acid residues, tryptophan and tyrosine exhibit different hydrophilic and hydrophobic properties.^[^ ^53^, ^56^ ^]^ Tryptophan and tyrosine are categorized as neutral in most hydrophobicity scales. It has previously been noted that tryptophan and tyrosine only express their hydrophilicity via a tiny CO(OH) or NH group in their long side‐chains, whereas the other portions of the side‐chains are highly hydrophobic alkyl and benzene ring structures (see Figure 2d).^[^ ^46^ ^]^ The characteristic of hydration shell water molecules surrounding hydrophobic groups is that their hydrogen bonding network is much more ordered than free liquid water molecules, that is, their entropy is much lower (less entropy in the system).^[^ ^57^, ^58^, ^59^, ^60^ ^]^ Therefore, ordered water molecules are fixed in the low‐entropy hydration shells around the highly hydrophobic alkyl and benzene ring structures and are expelled to van der Waals interaction operating distances. The single CO or NH group is most likely to hydrogen bond with the hydrophobic‐group‐induced low entropy water cages rather than destroy the ordered water molecule network (see Figure 2d). For instance, the hydrogen‐bond rearrangements between water molecules and the hydrophilic OH group of the tyrosine side chain can be inhibited by the hydrophobic benzene ring of the side chain because the OH group's neighboring water molecules are already fixed in the ordered network caused by the benzene ring. This explains why tryptophan and tyrosine are categorized as neutral or hydrophobic residues in hydrophobicity scales.^[^ ^54^, ^55^, ^56^ ^]^ Lysine also has a long side chain composed of a hydrophobic alkyl and one NH group. Twenty experimentally determined *α*‐helices are plotted in Figure S2 (Supporting Information) to illustrate that lysine can be involved in hydrophobic interactions with neighboring hydrophobic side chains in secondary structures. Therefore, the hydration shells surrounding the side chains of tryptophan, tyrosine, and lysine can be regarded as low‐entropy hydration shells (see Figure 2d); namely, we can consider the side chains to be pseudohydrophilic. When the side chains of tryptophan, tyrosine, and lysine are located at the surface of a protein, the hydration shells around them should be considered low‐entropy.

The hydrophilic group distribution on the protein surface before and after screening off the pseudohydrophilic groups is illustrated in Figure 2e and f. Another occasion for the pseudohydrophilic groups may be that a pair of positively or negatively charged side‐chains are located close to each other on the protein surfaces. The electrostatic repulsive side‐chains located close to each other are the result of protein folding, resulting in a decline in the polarity of the side‐chain pair, and the expression of the hydrophilicity may not be sufficient.

### 2.3 Mapping Low‐Entropy Regions of Hydration Shells of Proteins

**Figure 3:**
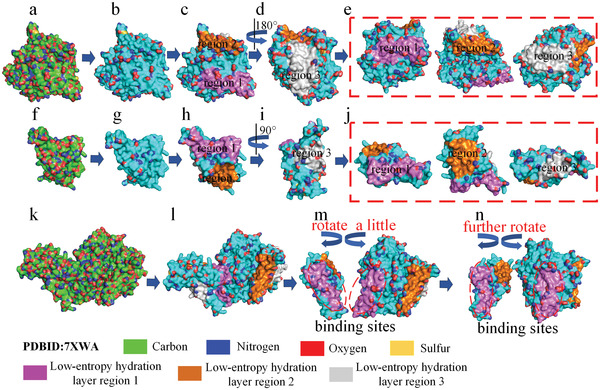
Low‐entropy hydration layer regions on the surfaces of ACE2 and RBD. a) ACE2. b) Distribution of low‐entropy hydration layer regions on the surface of ACE2. c–e) The three large low‐entropy regions of the hydration layer. f) The RBD. g) Distribution of low‐entropy hydration layer regions on the surface of the RBD. h–j) The three large low‐entropy regions of the hydration layer. k) RBD‐ACE2 complex. l) Distribution of the low‐entropy hydration layer region on the surfaces of the complex. m,n) The distribution of the low‐entropy hydration layer region on the binding sites of the complex.

Based on the above analysis, we obtained a method for screening off the pseudohydrophilic groups on a protein surface. After screening off the pseudohydrophilic groups, we mapped large low‐entropy regions of the hydration shell of a given protein (see Figure 2f). The shape and size matching between a large low‐entropy region of the hydration shell of the RBD of the spike protein of SARS‐CoV‐2 and a large low‐entropy region of the receptor angiotensin converting enzyme 2 (ACE2) is demonstrated in **Figure** **3**. The two low‐entropy regions cover the binding sites of the two proteins. The shape of a large low‐entropy hydration shell region of a given protein can be easily achieved from its front view. We mapped the large low‐entropy regions of the hydration shells of 700 proteins. Surprisingly, shape matching between one large low‐entropy hydration shell region of a protein and that of its partner at the binding sites prevailed in almost all the tested protein complexes in this study.

The shapes and sizes of large low‐entropy regions of protein hydration shells can be used as parameters to predict protein docking. By analyzing the hydrophobic attraction relationships among the low‐entropy regions of the protein hydration shells of the 700 protein complexes, we determined that the binding sites of a pair of proteins are always characterized by two rules of the spatial layout of the low‐entropy regions of the hydration shells at the binding sites. First, the docking position maximizes the overlap of one large low‐entropy hydration shell region of a protein and that of its partner. Second, the binding sites of a pair of proteins must allow sufficient interfacial contact at the docking position of the complex. Ordered water molecules fixed in the water cages of the low‐entropy regions of hydration shells that drive hydrophobic collapse of the low‐entropy hydration shell regions between proteins thereby rearrange ordered water molecules to free liquid water molecules to increase entropy. The binding affinity between two proteins is initially due to the long‐range hydrophobic effect among the low‐entropy hydration shell regions of the two proteins at the binding sites, enabling the shape‐matched large low‐entropy hydration shell regions to fully collapse between the two proteins.^[^ ^61^, ^62^, ^63^, ^64^ ^]^

### 2.4 Binding Sites Located in Large Low‐Entropy Regions and Verification with Test Systems

**Figure 4:**
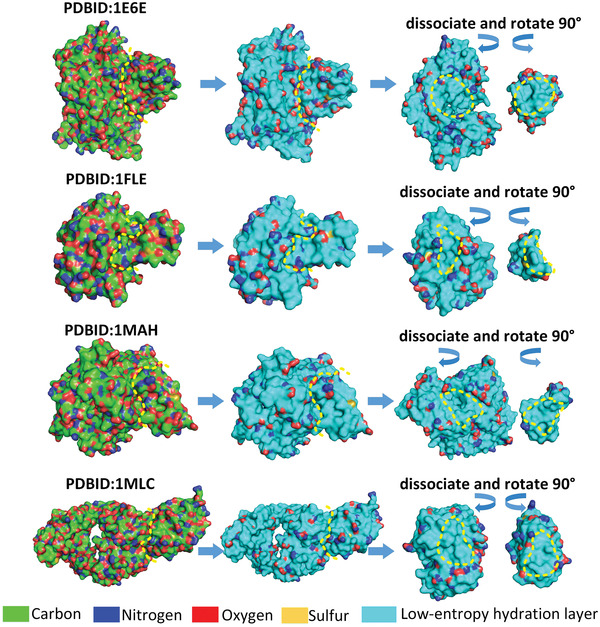
Low‐entropy regions of hydration shells covering the binding sites of proteins (from the protein‒protein docking benchmark version 4.0 test system). The binding sites of the two proteins are highlighted by yellow dashed lines, and the low‐entropy hydration shell region is highlighted in cyan. The remaining protein complexes of the test system are illustrated in the Supporting Information.

**Figure 5:**
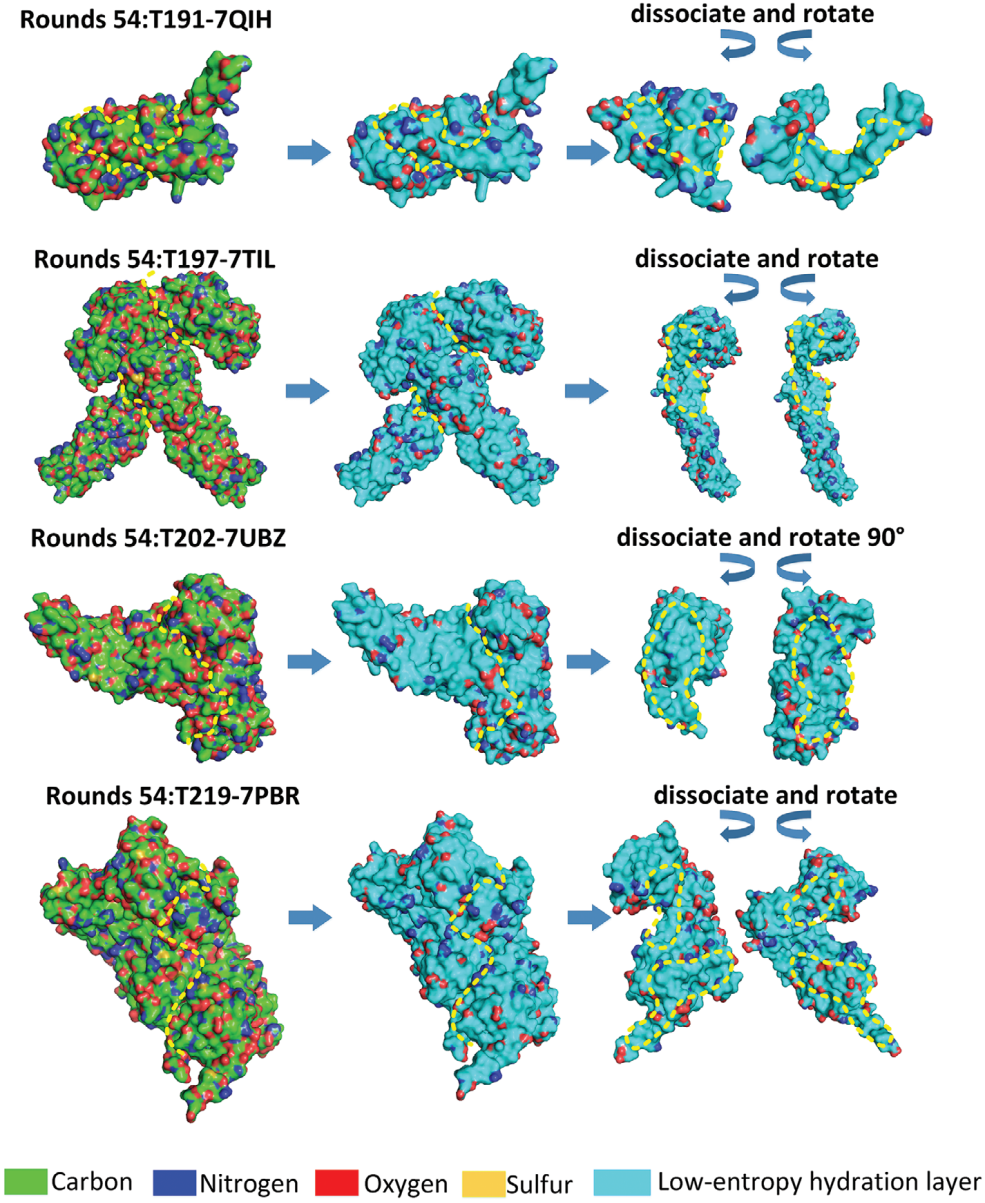
Low‐entropy regions of hydration shells covering the binding sites of proteins (test system CAPRI 54). The binding sites of the two proteins are highlighted by yellow dashed lines, and the low‐entropy hydration shell region is highlighted in cyan. The CAPRI target IDs and the corresponding PDBIDs are labeled.

A binding site of a protein should be one of the large low‐entropy hydration shell regions on the protein surface. The low‐entropy hydration shell regions of all 176 protein complexes of the test system of protein‒protein docking benchmark version 4.0 are mapped,^[^ ^65^ ^]^ as illustrated in **Figure** **4** and Figure S3 (Supporting Information). We found that the binding sites on the proteins are typically covered by large low‐entropy regions of the proteins’ hydration shells. Surprisingly, all the protein complexes of the test system show that the binding sites of proteins are the large low‐entropy regions on the proteins’ surfaces. This should be regarded as compelling evidence that protein‒protein binding is mainly guided by hydrophobic collapse between the shape‐matched low‐entropy regions on the proteins’ surfaces. A computer program is compiled to automatically screen off pseudohydrophilic groups and identify the low‐entropy hydration shell regions on the protein surfaces (see both sides of Figure S4, Supporting Information). Critical assessment of prediction of interactions (CAPRI) is a community‐wide initiative for testing computational algorithms in blind predictions of experimentally determined 3D structures of protein complexes. The round CAPRI 54 released 37 targets, and only four of the protein complex targets have been uploaded to the Protein Data Bank (PDB) thus far. The four protein complexes also show that the binding sites of proteins are large low‐entropy regions on the protein surfaces (see **Figure** **5**). Another 106 protein complexes randomly selected from the Protein Data Bank (PDB) are listed in the supplementary material to strengthen our verification (see Figure S4, Supporting Information).

**Figure 6:**
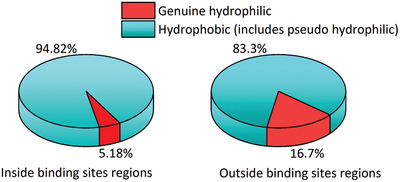
The average proportion of genuine hydrophilic atoms area to hydrophobic atoms area (including pseudohydrophilic atoms area) of the protein surfaces inside the binding site regions and outside the binding site regions. (Based on the Dockground test system).

We use another test system, Dockground, which contains 396 protein complexes, to further verify our low‐entropy hydration shell theory.^[^ ^66^ ^]^ We screened off surface pseudohydrophilic groups of all the individual proteins of the test system. We calculated the average proportion of genuine hydrophilic atoms to hydrophobic atoms (including pseudo hydrophilic atoms) of the protein surfaces inside the binding site regions and outside the binding site regions separately for all the proteins in the test system (see **Figure** **6** and Table S1, Supporting Information). Obviously, the proportion inside the binding site regions is much lower than that outside the binding site regions. This pattern almost prevailed in all the protein complexes (see Table S1, Supporting Information). Moreover, when an isolated hydrophilic group is surrounded by a low‐entropy hydration shell region at a protein surface, the hydration shell covering the hydrophilic group should be considered low‐entropy because the hydrophilic group's neighboring water molecules are already fixed in the ordered network.

### 2.5 Prediction of Binding Sites

**Figure 7:**
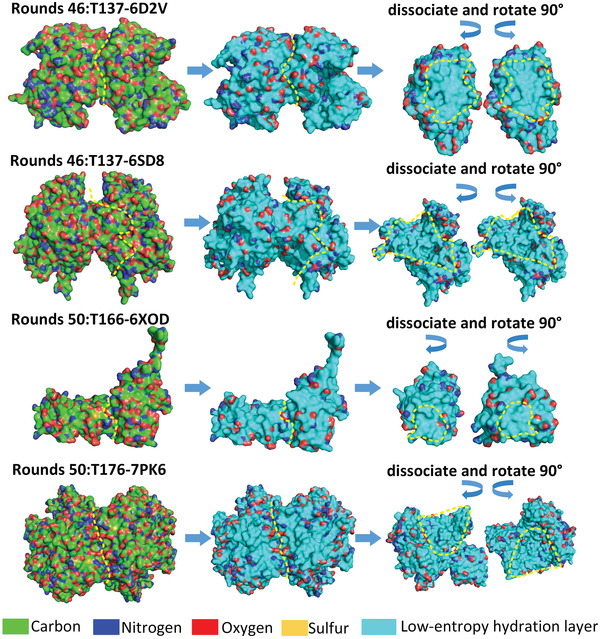
The prediction of binding sites of protein pairs from CAPRI rounds 46 and 50 test systems by identifying the shape‐matched low‐entropy regions of hydration shells of individual proteins. The remaining protein complexes of the test system are illustrated in Figure S4 (Supporting Information). The CAPRI target IDs and the corresponding PDBIDs are labeled. The binding sites of the two proteins are highlighted by yellow dashed lines, and the low‐entropy hydration shell region is highlighted in cyan.

To further prove that the protein‒protein binding process is guided by hydrophobic attraction between the shape‐matched large low‐entropy regions of hydration shells of the proteins, we tried to predict the binding sites of all 18 published protein complexes from another test system (CAPRI rounds 46 and 50) by using the above two rules (see **Figure** **7**; Figure S5, Supporting Information). All the binding sites of the 18 protein complexes were successfully predicted by using the two rules. The binding site is always one of the large low‐entropy regions of the hydration shell of a protein. All the samples are illustrated in the Supporting material. With further shape matches and hydrogen bonding matches, protein‒protein docking can most likely be accurately predicted.

**Figure 8:**
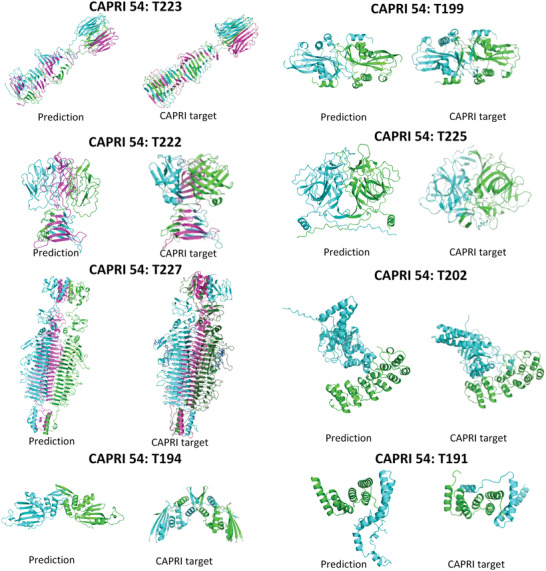
Prediction results submitted to the CAPRI 54. The CAPRI targets are plotted for the comparison . The three‐dimensional (3D) structure data of individual protein subunits is resourced from the Critical Assessment of Structure Prediction (CASP) prediction results generated by the Jumper group and Baker group. The CAPRI target IDs are labeled.

The prediction of binding sites of individual proteins promises prediction of the structures of the protein complexes, given the structures of the individual proteins. We participated in the CAPRI 54 by using the method of identifying low‐entropy regions of hydration shells of proteins. Comparison of our submitted prediction results to the CAPRI 54 and the CAPRI targets are illustrated in the **Figure** **8**. As shown in the Figure 8, the complex structures can be successfully and roughly predicted based on the method of maximization of the overlap of one large low‐entropy hydration shell region of a protein and that of its partner.^[^ ^67^ ^]^ With further electrostatic interaction and hydrogen bonding matches, protein‒protein docking can most likely be accurately predicted. The complexes predictions are based on the predicted tertiary structures of protein subunits generated by the Jumper group and Baker group.^[^ ^68^, ^69^ ^]^

### 2.6 Long‐Term Attracting Forces between Low‐Entropy Hydration Shells of Proteins

Hydrophobic interactions between two proteins in the binding process are physically powered by gradually removing ordered water molecules from the low‐entropy hydration shells at the binding site of the individual protein, which increases the entropy. The hydration shell (that is, the hydration layer) around a protein has been found to have dynamics distinct from those of bulk water to a distance of ≈5 nm.^[^ ^19^, ^32^, ^37^ ^]^ Thus, the standard molar entropy of water within the ordered cages (that is, the hydration shells) around the nonpolar surface is approximately equal to the standard molar entropy of solid water, which is ≈41 J mol K^−1^. The standard molar entropy of liquid water is ≈70 J mol K^−1^.^[^ ^70^ ^]^ Therefore, moving an ordered water molecule from a low‐entropy hydration shell to a free liquid result in a molar entropy difference ∆S of ≈29 J mol K^−1^. At a human body temperature of T = 309 K, the free energy increment is T∆S = 8961 J mol^−1^ for one water molecule with a removed hydration shell. Therefore, at T = 309 K, the binding force between ACE2 and the RBD at a distance of 10 nm is ≈4.37 nN.^[^ ^44^, ^71^ ^]^ Obviously, hydrophobic interactions between the two proteins can provide enough binding force to drew the coronavirus approaching the recipient cell because each coronavirus has a mass of 1 femtogram. Experimental results have revealed that at a larger separation distance of two monomers (≈5 nm), the dynamic properties of confined water molecules exhibit considerable deviation from bulk‐like characteristics.^[^ ^32^ ^]^ This indicates that strong hydrophobic interactions between two proteins most likely have a starting distance greater than 10 nm.

## 3 Conclusion 

It is worth noting that some protein subunites are dynamic molecules with extensive conformational flexibility at the binding sites. If prediction of the binding sites is based on a flexible view of protein structure, searching protein docking results is limited and high computational cost. Nevertheless, the distribution of hydrophbic groups at binding sites of proteins is fixed by the surface topography. Thus, the low‐entropy hydration shell at the binding site of a protein subunit most likely spatial approximate match with that of the receptor protein at a certain time, make the two binding sites approaching to each other due to the hydrophobic interaction. Due to convenient interface fitting between the two binding sites and hydrophobic interaction between strong hydrophobic groups from both proteins, conformational flexibility of protein at the binding sites most likely is beneficial to the binding process. Moreover, it is possible for intrinsically disordered proteins to contain hydrophobic groups at some fragments, resulting in the possibility of forming low‐entropy hydration shells at the fragments. As a result, some disordered proteins can bind with rigid proteins to form stable complex structures. While protein binding sites can be approximately predicted by mapping the low‐entropy regions, protein configurational flexibility should also be considered to accurately predict PPIs.

As a typical spontaneous reaction, the approaching stage of protein‒protein binding must release Gibbs free energy as it proceeds, but not be dominated by electrostatic interaction or hydrogen bonding between the two proteins due to the shielding effect of the polar water molecules of the hydration shells of individual proteins. When two paired proteins bind, their hydration shells must gradually be squeezed out at the binding sites by a physical mechanism. The reasons that the hydrophilic groups on protein surfaces do not express their hydrophilicity are summarized. The large low‐entropy regions of hydration shells of given proteins can be mapped by screening off the pseudohydrophilic groups on protein surfaces. By the analysis, we show that the binding sites of protein pairs were typically covered by the larger shape‐matched low‐entropy regions of the hydration shells, enabling the large low‐entropy regions of hydration shells to fully hydrophobic collapse in the binding process. The entropy increase caused by hydrophobic attraction guides the docking process and provides binding affinity. Despite the difficulty in identifying low‐entropy hydration shells around a protein using experimental methods, theoretical approaches allow us to map large low‐entropy regions of hydration shells around proteins that can be used to accurately predict protein binding sites. The spatial layout of the low‐entropy region of the protein's hydration shell acts like a “lock and key” for guiding protein‒protein binding in a precise manner.

## 4 Experimental Section

### Protein Structures

In this study, it was used numerous experimentally determined protein structures to study protein‐protein docking mechanism. All the 3D structure data of protein molecules were obtained from the Protein Data Bank (PDB).^[^ ^72^ ^]^ IDs of these proteins according to the PDB database were labeled in all the figures. To show the distribution of low‐entropy hydration shells on the surface of proteins at the binding sites in these figures, the structural biology visualization software PyMOL to display the low‐entropy hydration shell areas was used.^[^ ^73^ ^]^ The list of PDB files of the test systems was illustrated in protein–protein docking benchmark version 4.0.^[^ ^65^ ^]^

### Hydrophilicity of Residues

The topological structures in charmm36 force field describe in detail of all atomic charge amount and spatial conformations of amino acids, which can be used to identify hydrophobicity and hydrophilicity of residues (see Figure S1, Supporting Information).^[^ ^74^ ^]^ For example, the charge amounts of carbon atoms on residue sidechains mainly range from −0.27 to −0.09e and make it difficult to form hydrogen bonds with water molecules, a phenomenon known as hydrophobicity. Conversely, the larger electronegativity of nitrogen atoms (mainly from −0.47 to −0.8e) and oxygen atoms (from −0.51 to −0.76) allows these atoms to form hydrogen bonds with surrounding water molecules, a phenomenon known as hydrophilicity. When hydrophilic groups on protein surface in aqueous solution make favorable interior hydrogen bonds with each other but not with water molecules (the charge amount of oxygen atom in TIP3P model was −0.834e), it would argue that the hydration shells of hydrophilic groups were also low‐entropy.

### Prediction of the Binding Sites

Protein surface hydration dynamics were not simply determined by the distribution of hydrophilic and hydrophobic groups on the protein surface.^[^ ^31^, ^75^ ^]^ Some hydrophilic groups on protein surfaces that do not express their hydrophilicity. A custom Python script was compiled to automatically screen off pseudohydrophilic groups and identify the low‐entropy hydration shell regions on the protein surfaces via three steps. First, when hydrophilic groups (related to oxygen, nitrogen toms) on protein surface form stable intramolecular hydrogen bonds with each other, it was consider that the hydration shell of the hydrophilic groups was also low‐entropy. The intramolecular hydrogen bonds can be identified by using PyMOL. Second, When the side chains of isoleucine, valine, leucine, tyrosine, tryptophan, cysteine, methionine, phenylalanine, lysine, arginine, histidine, proline, and alanine residues, containing highly hydrophobic structures, were located on the protein surface and protrude outward to surrounding water molecules, which can shield the hydrophilic backbone carbonyl oxygen atoms and amide hydrogen atoms to forming hydrogen bonds with water molecules. Thus, the hydration shells of backbone carbonyl oxygen atoms and amide hydrogen atoms of these above residues were considered as low‐entropy. Third, the hydration shells of the CO and NH groups of tryptophan, tyrosine, and lysine should also be low‐entropy because of long hydrophobic chain. On the protein surface, only the rest of the hydrophilic groups can normally express their hydrophilicity. On a protein surface, after screening off pseudohydrophilic groups, genuine hydrophilic groups were no longer distributed throughout the surface, several regions on the surface were only composed of hydrophobic groups and pseudohydrophilic groups (see Figure 3). As a result, that can consider these regions to be low‐entropy regions of the protein's hydration shell. Therefore, low‐entropy regions of the hydration shell of a protein can be mapped by using the method of screening off pseudohydrophilic groups on protein surfaces.

Based on the above results, the low‐entropy regions of the hydration shells of the two proteins are plotted in Figure 2, Figure 3, Figure 4, and Figures S4 and S6 (Supporting Information). Although protein subunits have more than one large low‐entropy region on their surface, two shape‐matched low‐entropy hydration regions on individual proteins can be only identified. The binding sites of the two paired proteins are thus successfully predicted. In summary, protein structures were taken into account for testing our hypothesis include protein‒protein docking benchmark version 4.0 (176 protein complexes),^[^ ^65^ ^]^ randomly selected 106 protein complexes in PDB database,^[^ ^72^ ^]^ Dockground database (396 protein complexes)^[^ ^66^ ^]^ and 22 CAPRI 46,50,54 target protein complexes.

## Conflict of Interest

The authors declare no conflict of interest.

## Author Contributions

L.Yang, L.Ye, and X.H. formulated the study. L.Yang, S.G., C.H., L.S., J.S., X.M. and C.L. collected and analyzed the PDB data. L.Yang, C.L., C.H. wrote programs. L.Yang, and X.H. wrote the paper, and all authors contributed to revising it. All authors discussed the results and theoretical interpretations.

## Additional Information

The authors declare the methods used in this paper have been protected by our patents, such as, the Chinese Patent No: 202 210 138 581.X. and America patent and European patent “Method and device for protein‐protein docking based on identification of low‐entropy hydration layer on protein surface”.

## Supporting information

Supporting Information

## Data Availability

The data that support the findings of this study are available in the supplementary material of this article.
