# Supplementary material for: Spatial Layouts of Low‐Entropy Hydration Shells Guide Protein Binding
Source: Glob Chall. 2023 May 2;7(7):2300022. doi: 10.1002/gch2.202300022 (PMC10362119; doi:10.1002/gch2.202300022)
Supplement: Supplementary file 1 — Supporting Information [file GCH2-7-2300022-s001.pdf]

Global Challenges
Open Access

Supporting Information

for *Global Challenges.*, DOI 10.1002/gch2.202300022

Spatial Layouts of Low-Entropy Hydration Shells Guide Protein Binding

Lin Yang, Shuai Guo, Chenchen Liao, Chengyu Hou, Shenda Jiang, Jiacheng Li, Xiaoliang Ma, Liping Shi, Lin Ye and Xiaodong He**

Supplementary information

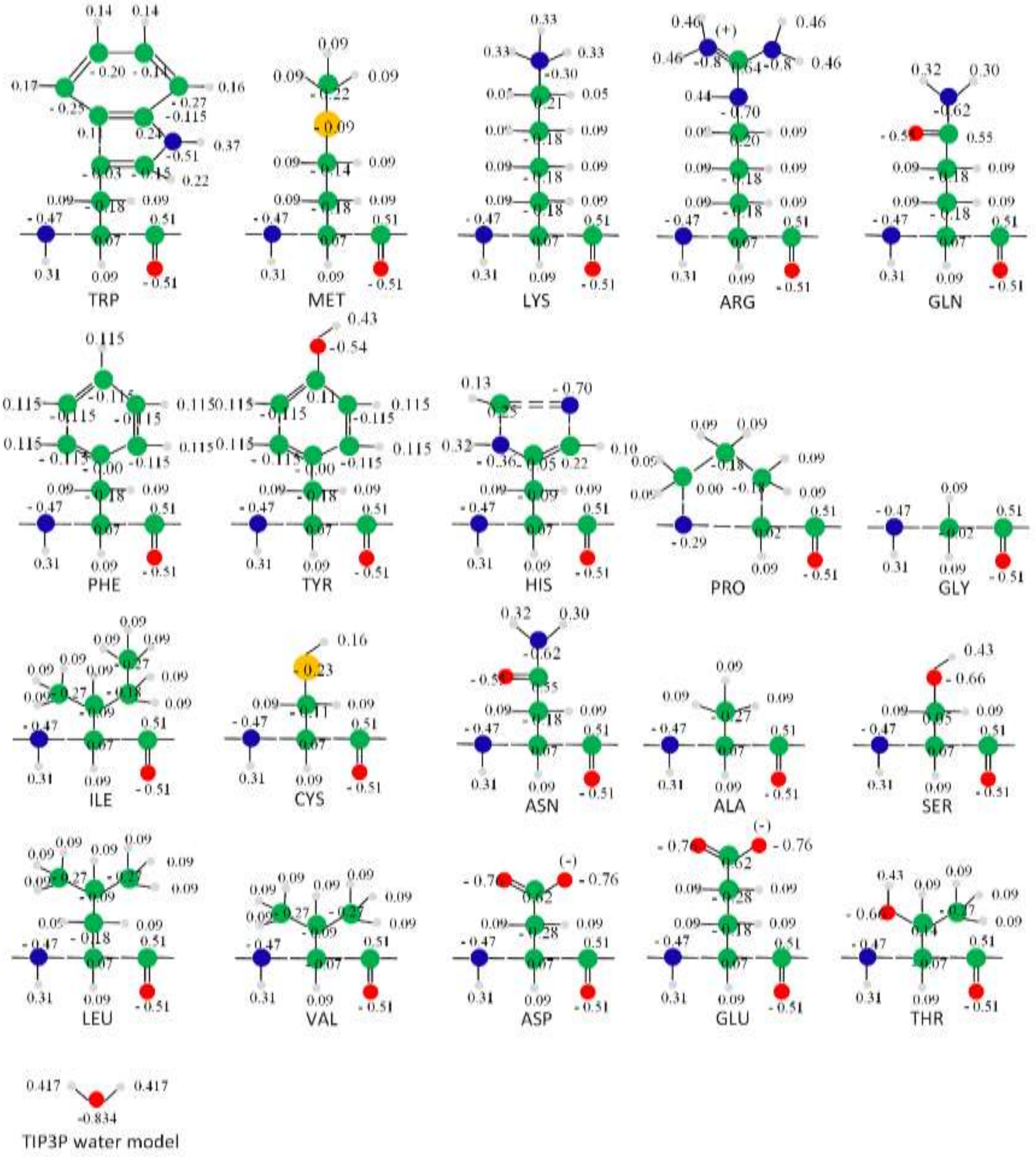

Fig.S1 The atomic charge amounts of residues according to the charmm36 force field. Carbon atoms, sulphur atoms and their hydrogen-bonded hydrogen atoms should be considered hydrophobic, according to this figure.

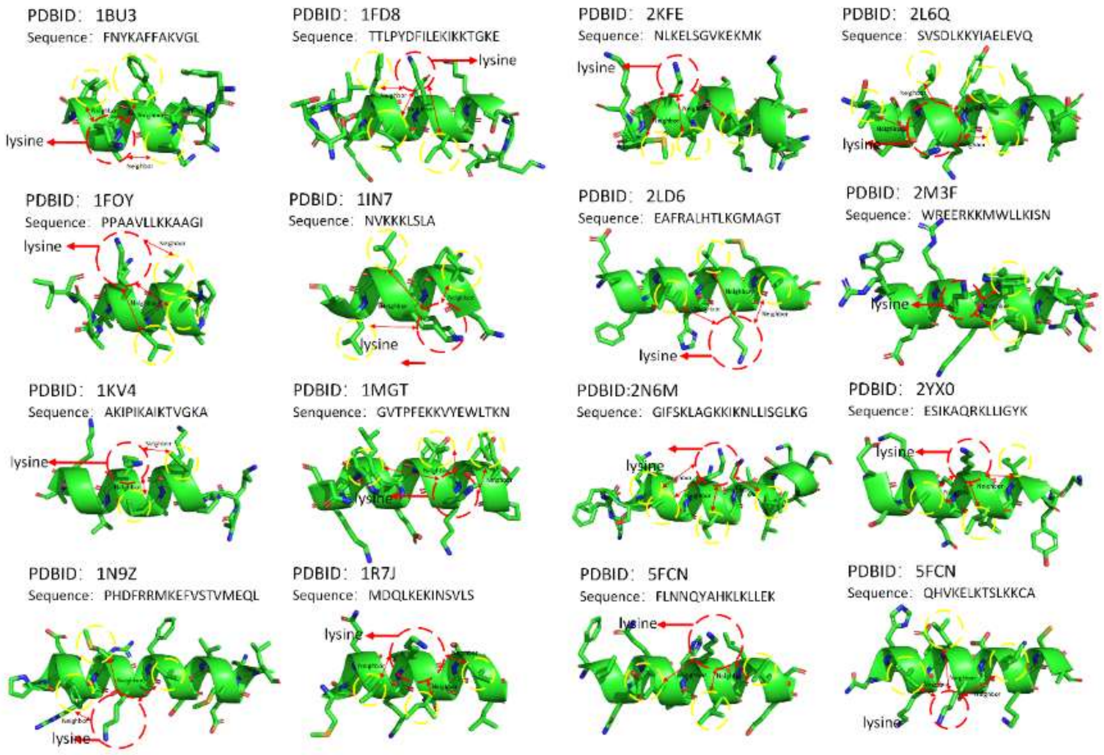

Fig.S2 Lysine involved in hydrophobic interaction with neighbored hydrophobic side-chains in the α-helices of the proteins selected form PDB. Hydrophobic side-chains are highlighted by yellow dash circles.

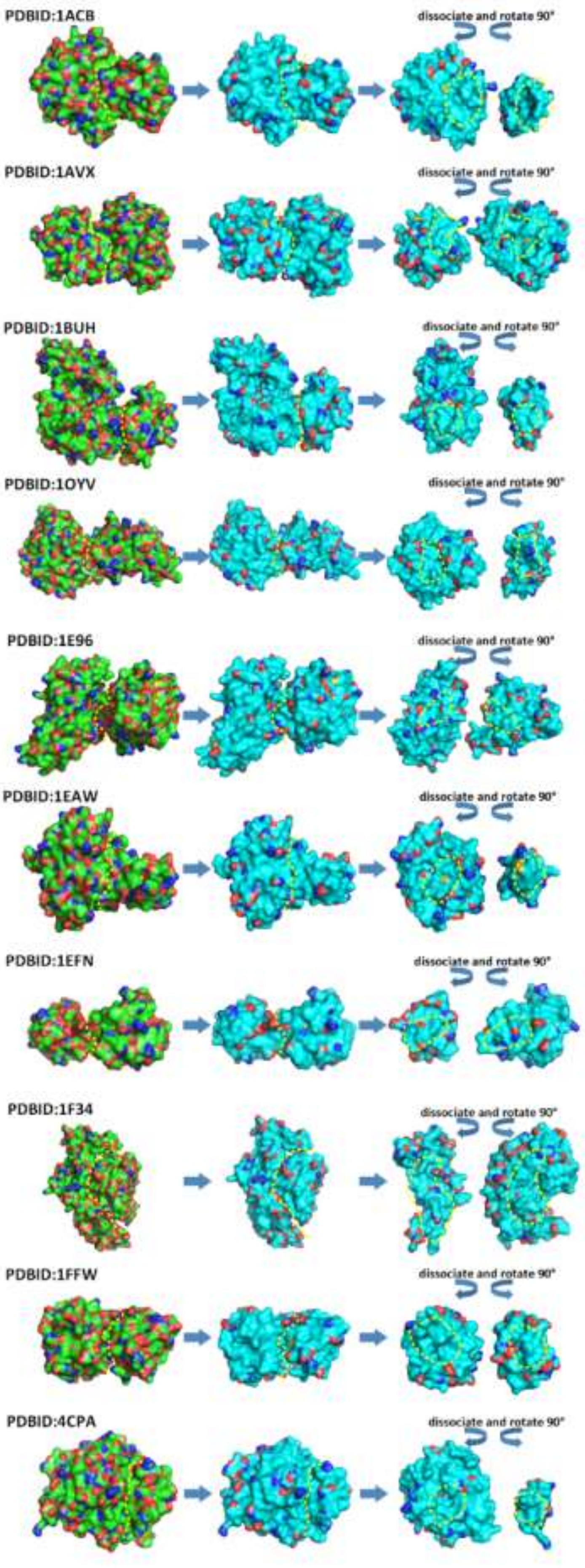
PDBID:1ACB
dissociate and rotate 90°
PDBID:1AVX
dissociate and rotate 90°
PDBID:1BUH
dissociate and rotate 90°
PDBID:1OYV
dissociate and rotate 90°
PDBID:1E96
dissociate and rotate 90°
PDBID:1EAW
dissociate and rotate 90°
PDBID:1EFN
dissociate and rotate 90°
PDBID:1F34
dissociate and rotate 90°
PDBID:1FFW
dissociate and rotate 90°
PDBID:4CPA
dissociate and rotate 90°

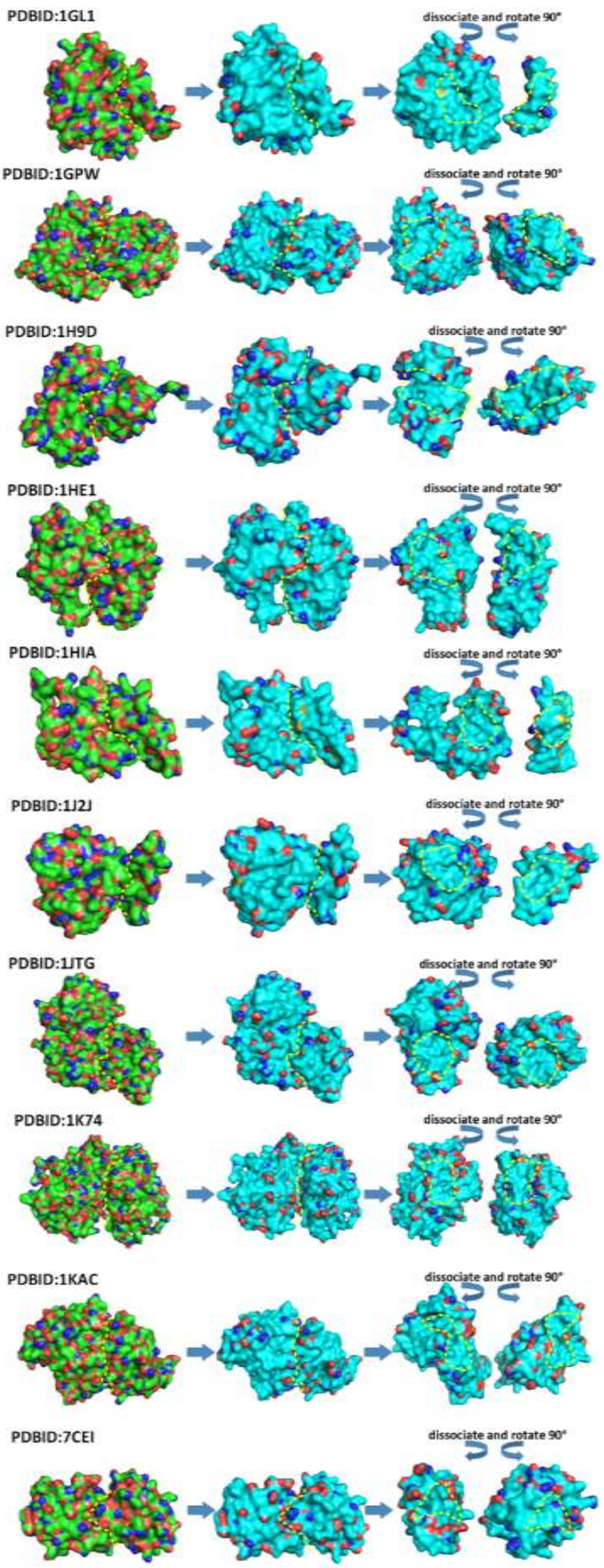
PDBID:1GL1
dissociate and rotate 90°
PDBID:1GPW
dissociate and rotate 90°
PDBID:1H9D
dissociate and rotate 90°
PDBID:1HE1
dissociate and rotate 90°
PDBID:1HIA
dissociate and rotate 90°
PDBID:1J2J
dissociate and rotate 90°
PDBID:1JTG
dissociate and rotate 90°
PDBID:1K74
dissociate and rotate 90°
PDBID:1KAC
dissociate and rotate 90°
PDBID:7CEI
dissociate and rotate 90°

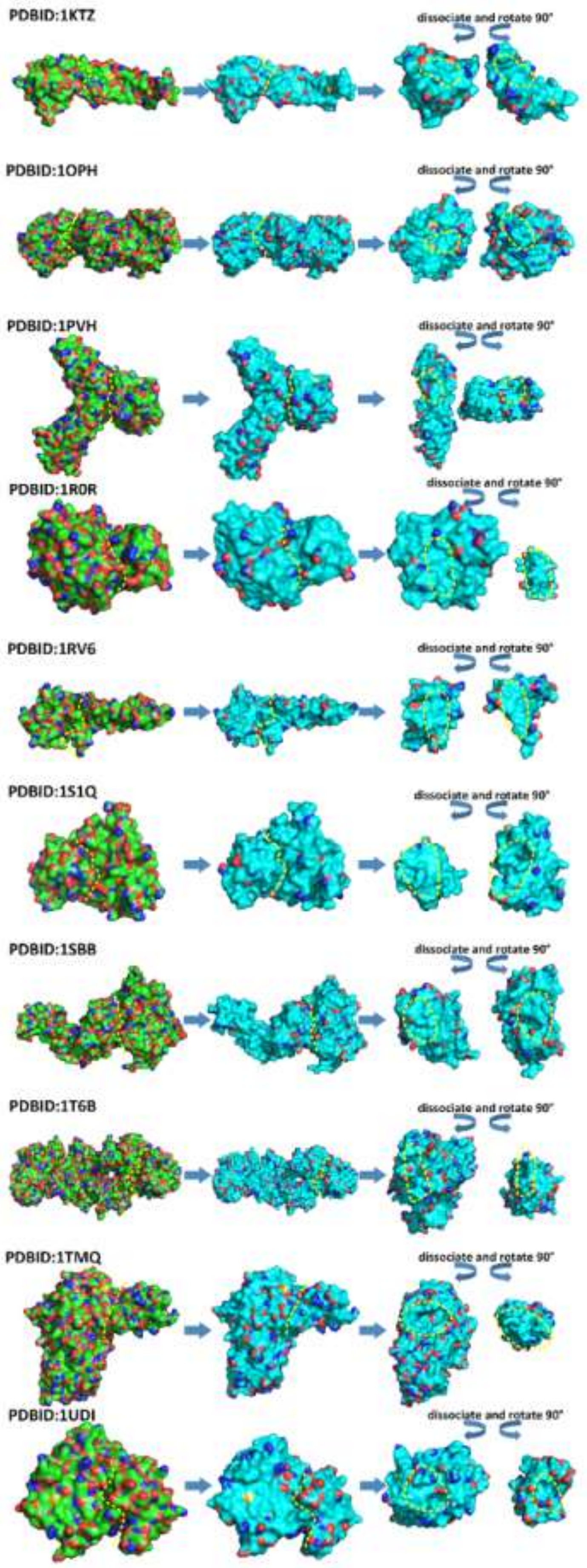
PDBID:1KTZ
dissociate and rotate 90°
PDBID:1OPH
dissociate and rotate 90°
PDBID:1PVH
dissociate and rotate 90°
PDBID:1R0R
dissociate and rotate 90°
PDBID:1RV6
dissociate and rotate 90°
PDBID:1S1Q
dissociate and rotate 90°
PDBID:1SBB
dissociate and rotate 90°
PDBID:1T6B
dissociate and rotate 90°
PDBID:1TMQ
dissociate and rotate 90°
PDBID:1UDI
dissociate and rotate 90°

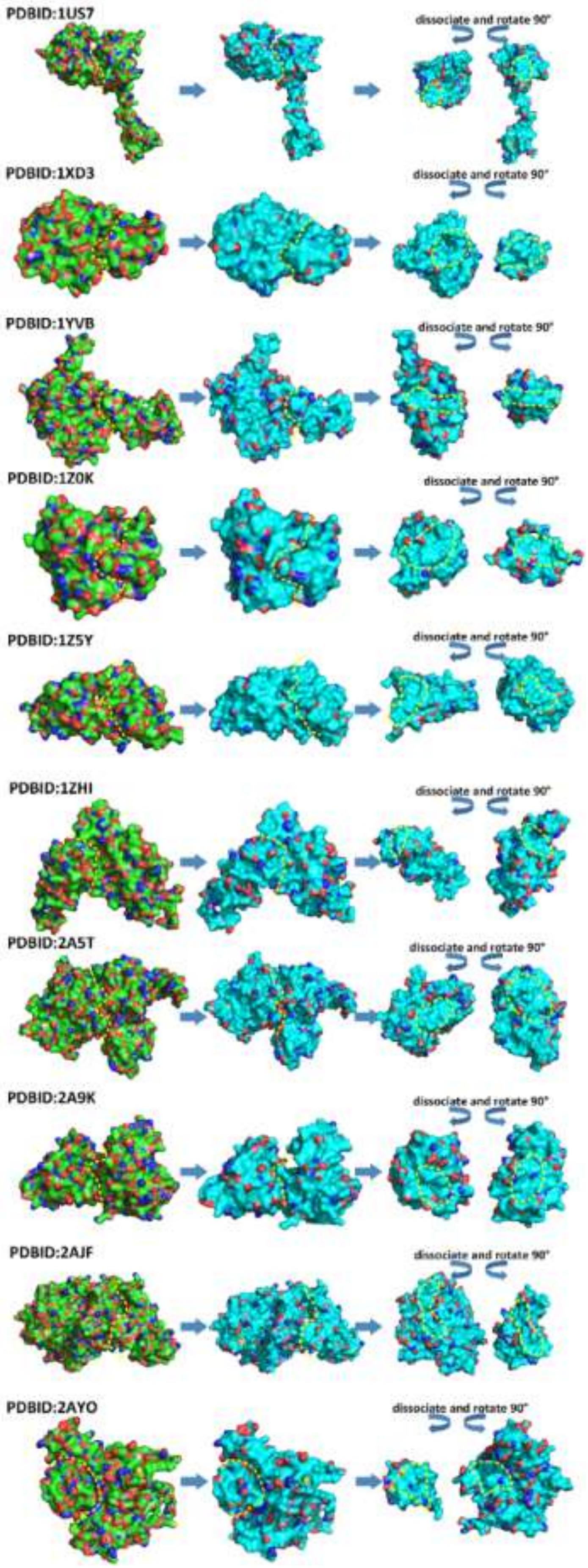
PDBID:1US7
dissociate and rotate 90°
PDBID:1XD3
dissociate and rotate 90°
PDBID:1YVB
dissociate and rotate 90°
PDBID:1Z0K
dissociate and rotate 90°
PDBID:1Z5Y
dissociate and rotate 90°
PDBID:1ZHI
dissociate and rotate 90°
PDBID:2A5T
dissociate and rotate 90°
PDBID:2A9K
dissociate and rotate 90°
PDBID:2AJF
dissociate and rotate 90°
PDBID:2AYO
dissociate and rotate 90°

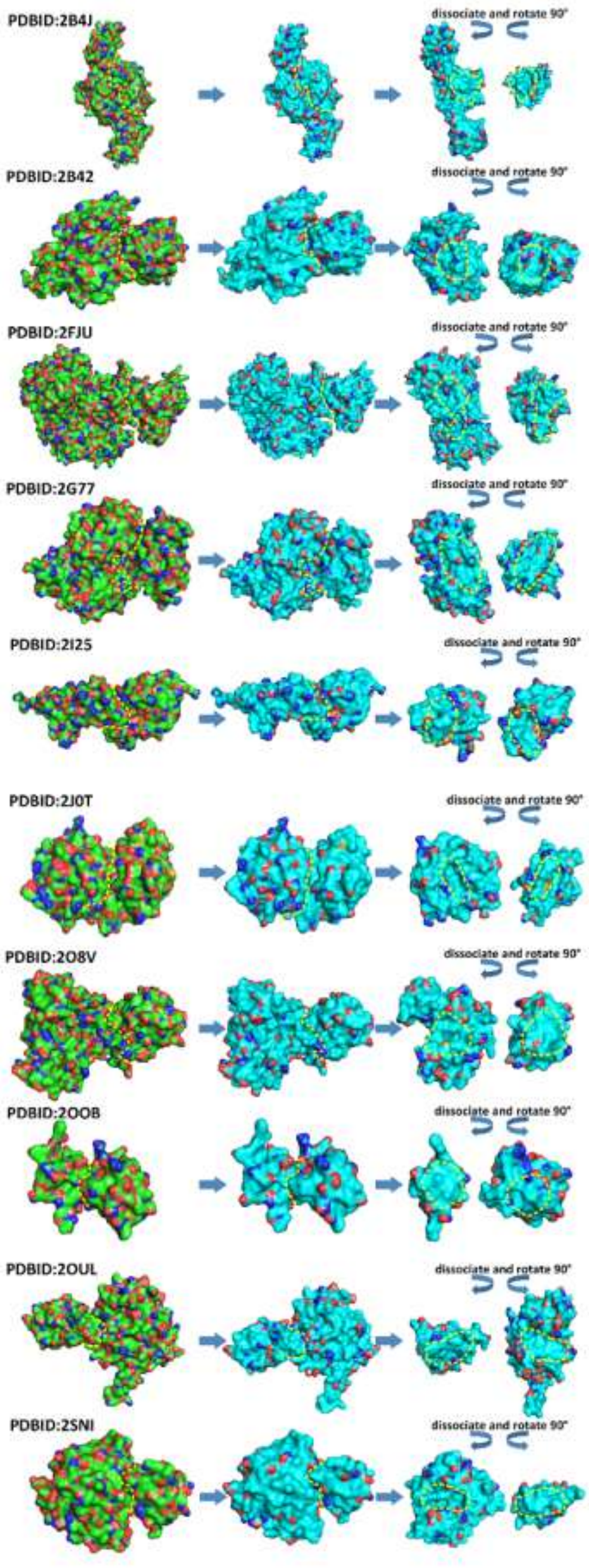
PDBID:2B4J
dissociate and rotate 90°
PDBID:2B42
dissociate and rotate 90°
PDBID:2FJU
dissociate and rotate 90°
PDBID:2G77
dissociate and rotate 90°
PDBID:2I25
dissociate and rotate 90°
PDBID:2J0T
dissociate and rotate 90°
PDBID:2O8V
dissociate and rotate 90°
PDBID:2OOB
dissociate and rotate 90°
PDBID:2OUL
dissociate and rotate 90°
PDBID:2SNI
dissociate and rotate 90°

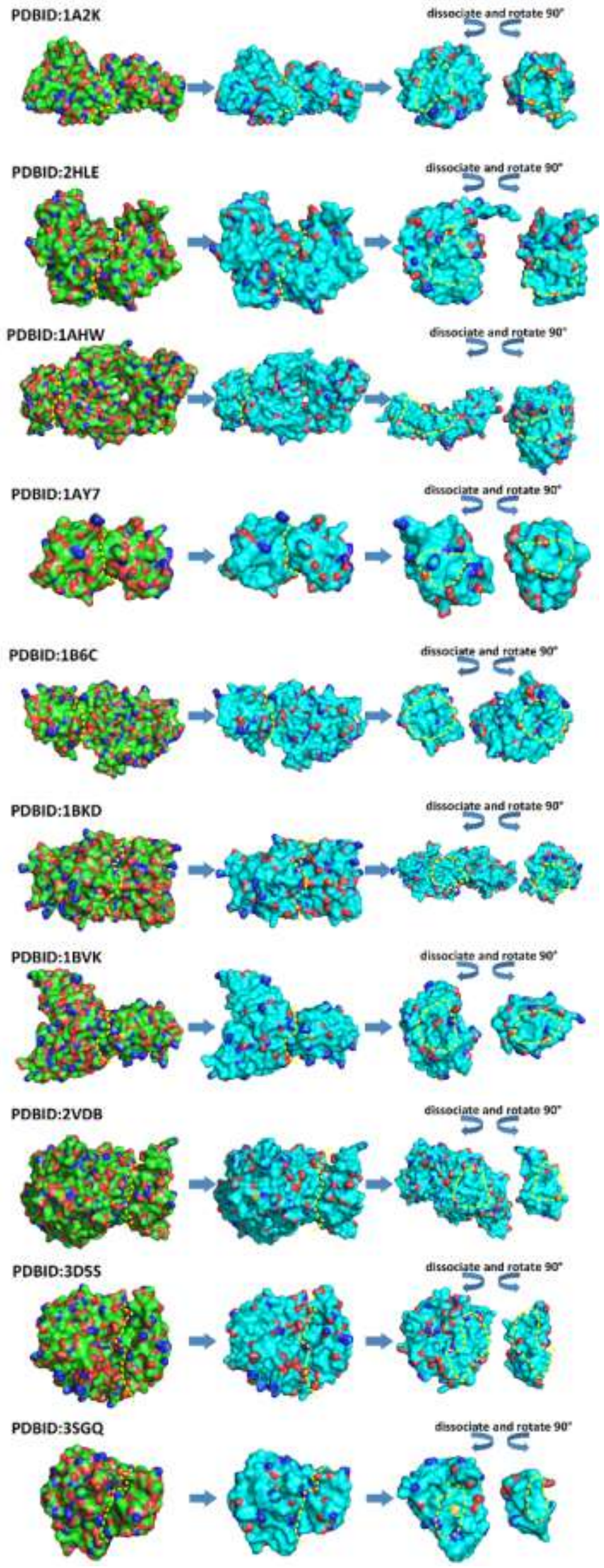
PDBID:1A2K
dissociate and rotate 90°
PDBID:2HLE
dissociate and rotate 90°
PDBID:1AHW
dissociate and rotate 90°
PDBID:1AY7
dissociate and rotate 90°
PDBID:1B6C
dissociate and rotate 90°
PDBID:1BKD
dissociate and rotate 90°
PDBID:1BVK
dissociate and rotate 90°
PDBID:2VDB
dissociate and rotate 90°
PDBID:3D5S
dissociate and rotate 90°
PDBID:3SGQ
dissociate and rotate 90°

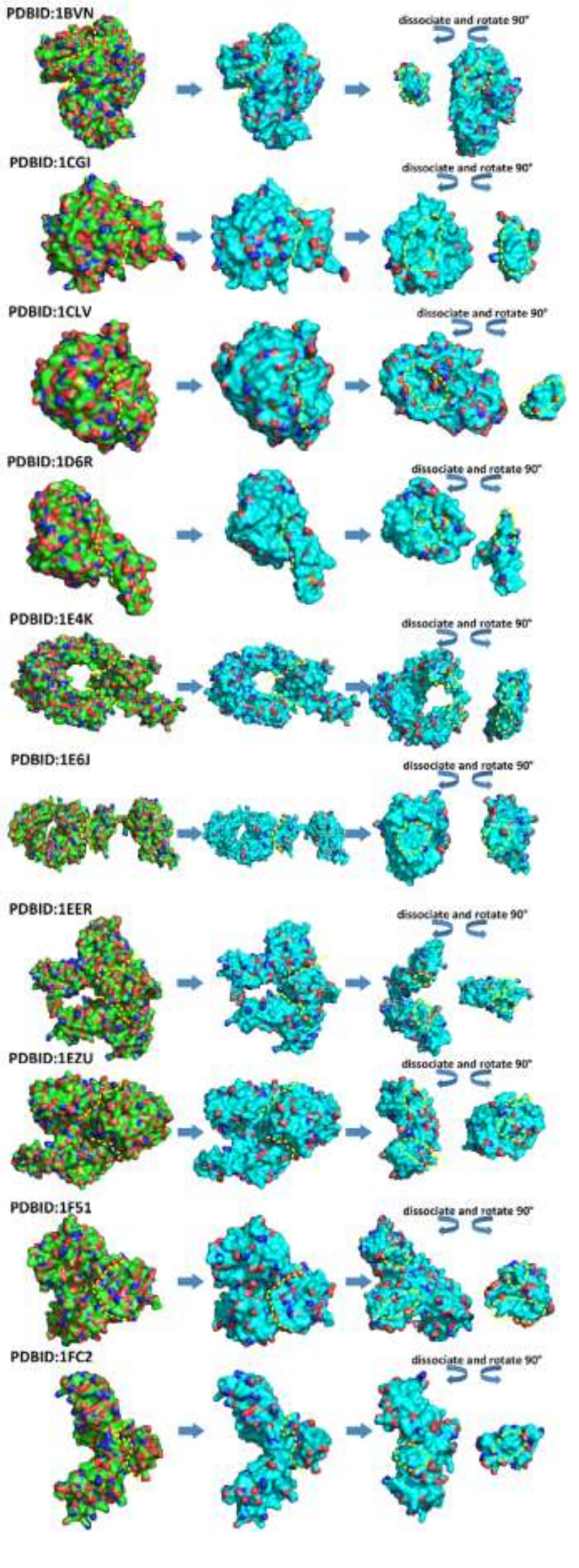

PDBID:1BVN
dissociate and rotate 90°
PDBID:1CGI
dissociate and rotate 90°
PDBID:1CLV
dissociate and rotate 90°
PDBID:1D6R
dissociate and rotate 90°
PDBID:1E4K
dissociate and rotate 90°
PDBID:1E6J
dissociate and rotate 90°
PDBID:1EER
dissociate and rotate 90°
PDBID:1EZU
dissociate and rotate 90°
PDBID:1F51
dissociate and rotate 90°
PDBID:1FC2
dissociate and rotate 90°

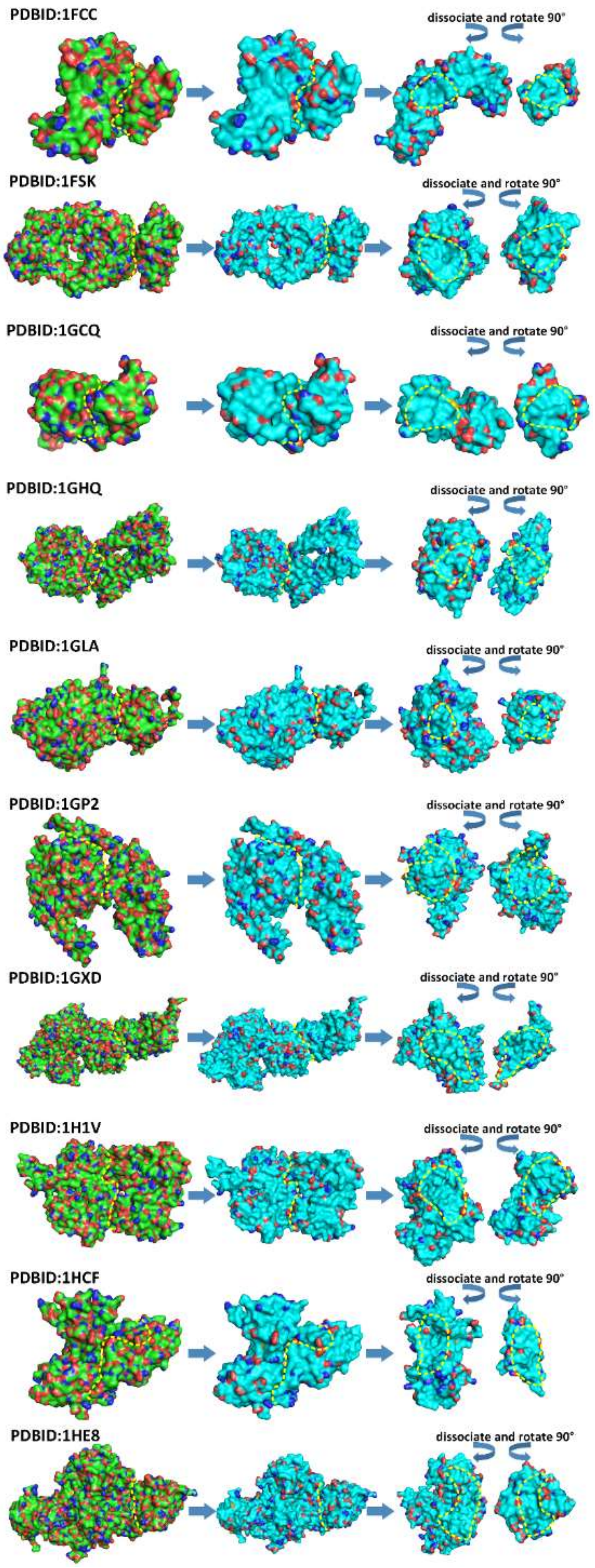
PDBID:1FCC
dissociate and rotate 90°
PDBID:1FSK
dissociate and rotate 90°
PDBID:1GCQ
dissociate and rotate 90°
PDBID:1GHQ
dissociate and rotate 90°
PDBID:1GLA
dissociate and rotate 90°
PDBID:1GP2
dissociate and rotate 90°
PDBID:1GXD
dissociate and rotate 90°
PDBID:1H1V
dissociate and rotate 90°
PDBID:1HCF
dissociate and rotate 90°
PDBID:1HE8
dissociate and rotate 90°

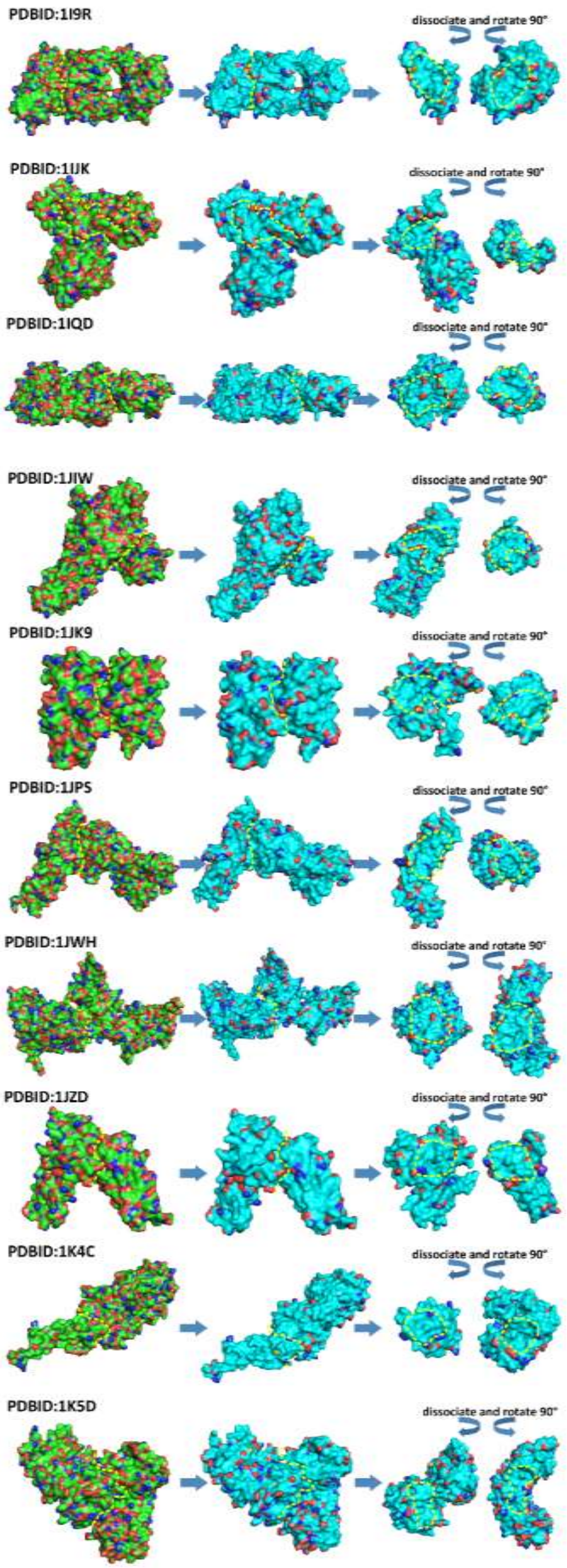
PDBID:1I9R
dissociate and rotate 90°
PDBID:1IJK
dissociate and rotate 90°
PDBID:1IQD
dissociate and rotate 90°
PDBID:1JIW
dissociate and rotate 90°
PDBID:1JK9
dissociate and rotate 90°
PDBID:1JPS
dissociate and rotate 90°
PDBID:1JWH
dissociate and rotate 90°
PDBID:1JZD
dissociate and rotate 90°
PDBID:1K4C
dissociate and rotate 90°
PDBID:1K5D
dissociate and rotate 90°

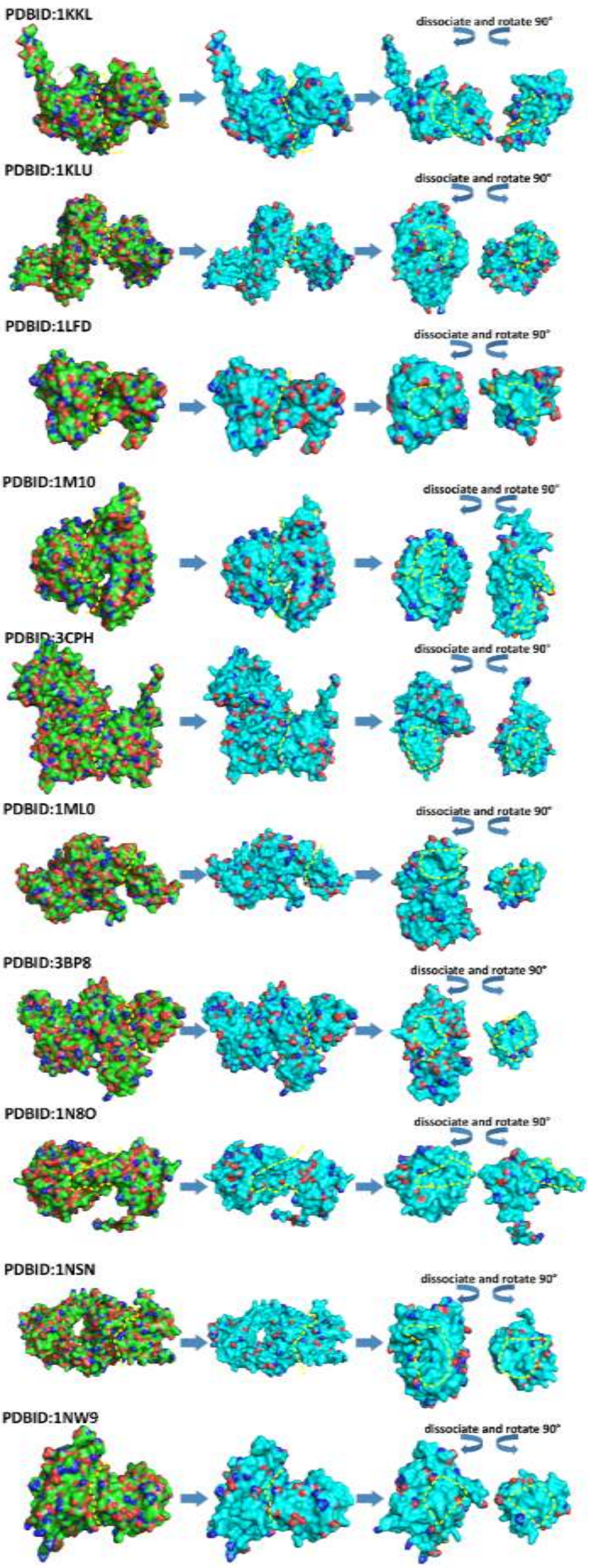
PDBID:1KKL
dissociate and rotate 90°
PDBID:1KLU
dissociate and rotate 90°
PDBID:1LFD
dissociate and rotate 90°
PDBID:1M10
dissociate and rotate 90°
PDBID:3CPH
dissociate and rotate 90°
PDBID:1ML0
dissociate and rotate 90°
PDBID:3BP8
dissociate and rotate 90°
PDBID:1N8O
dissociate and rotate 90°
PDBID:1NSN
dissociate and rotate 90°
PDBID:1NW9
dissociate and rotate 90°

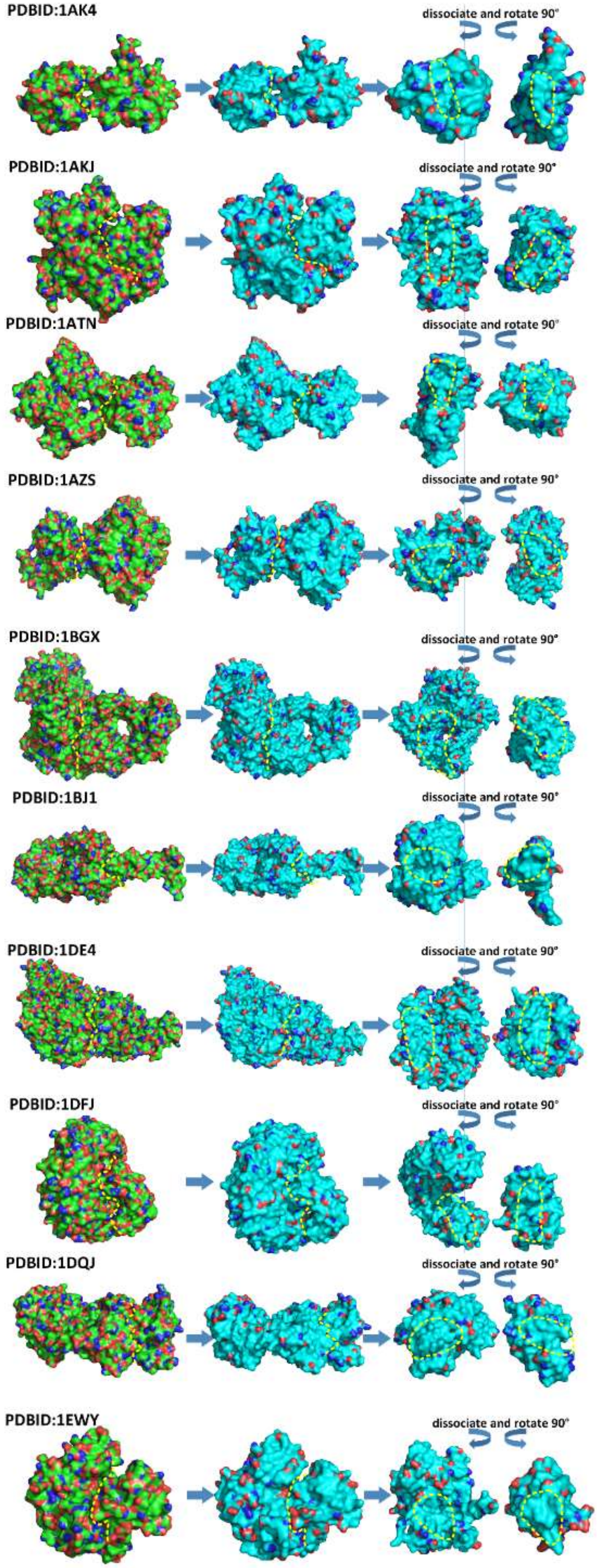
PDBID:1AK4
dissociate and rotate 90°
PDBID:1AKJ
dissociate and rotate 90°
PDBID:1ATN
dissociate and rotate 90°
PDBID:1AZS
dissociate and rotate 90°
PDBID:1BGX
dissociate and rotate 90°
PDBID:1BJ1
dissociate and rotate 90°
PDBID:1DE4
dissociate and rotate 90°
PDBID:1DFJ
dissociate and rotate 90°
PDBID:1DQJ
dissociate and rotate 90°
PDBID:1EWY
dissociate and rotate 90°

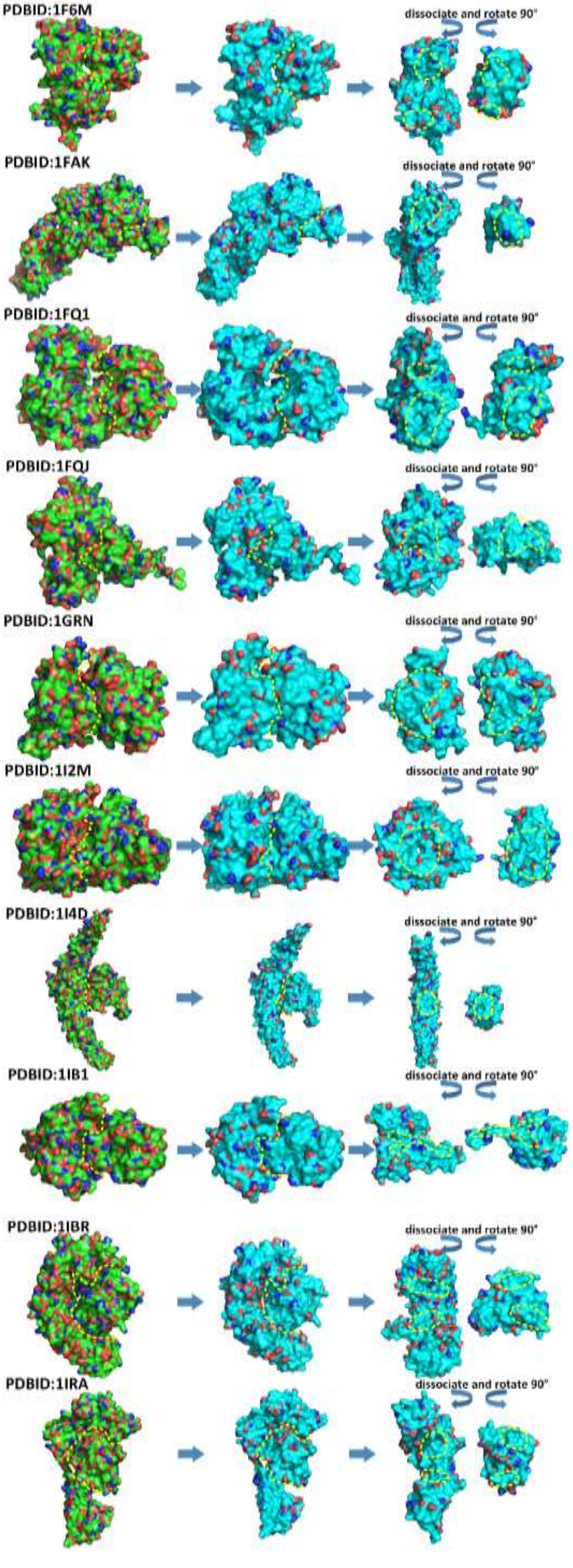
PDBID:1F6M
dissociate and rotate 90°
PDBID:1FAK
dissociate and rotate 90°
PDBID:1FQ1
dissociate and rotate 90°
PDBID:1FQJ
dissociate and rotate 90°
PDBID:1GRN
dissociate and rotate 90°
PDBID:1I2M
dissociate and rotate 90°
PDBID:1I4D
dissociate and rotate 90°
PDBID:1IB1
dissociate and rotate 90°
PDBID:1IBR
dissociate and rotate 90°
PDBID:1IRA
dissociate and rotate 90°

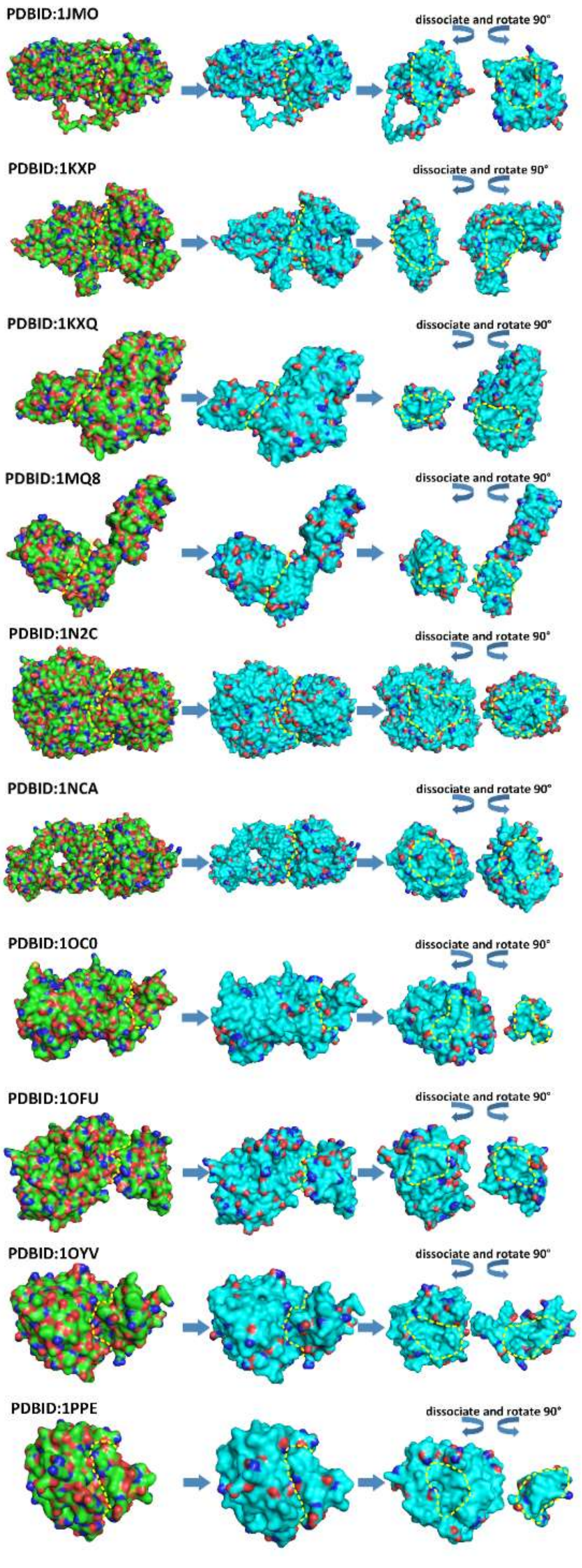
PDBID:1JMO
dissociate and rotate 90°
PDBID:1KXP
dissociate and rotate 90°
PDBID:1KXQ
dissociate and rotate 90°
PDBID:1MQ8
dissociate and rotate 90°
PDBID:1N2C
dissociate and rotate 90°
PDBID:1NCA
dissociate and rotate 90°
PDBID:1OC0
dissociate and rotate 90°
PDBID:1OFU
dissociate and rotate 90°
PDBID:1OYV
dissociate and rotate 90°
PDBID:1PPE
dissociate and rotate 90°

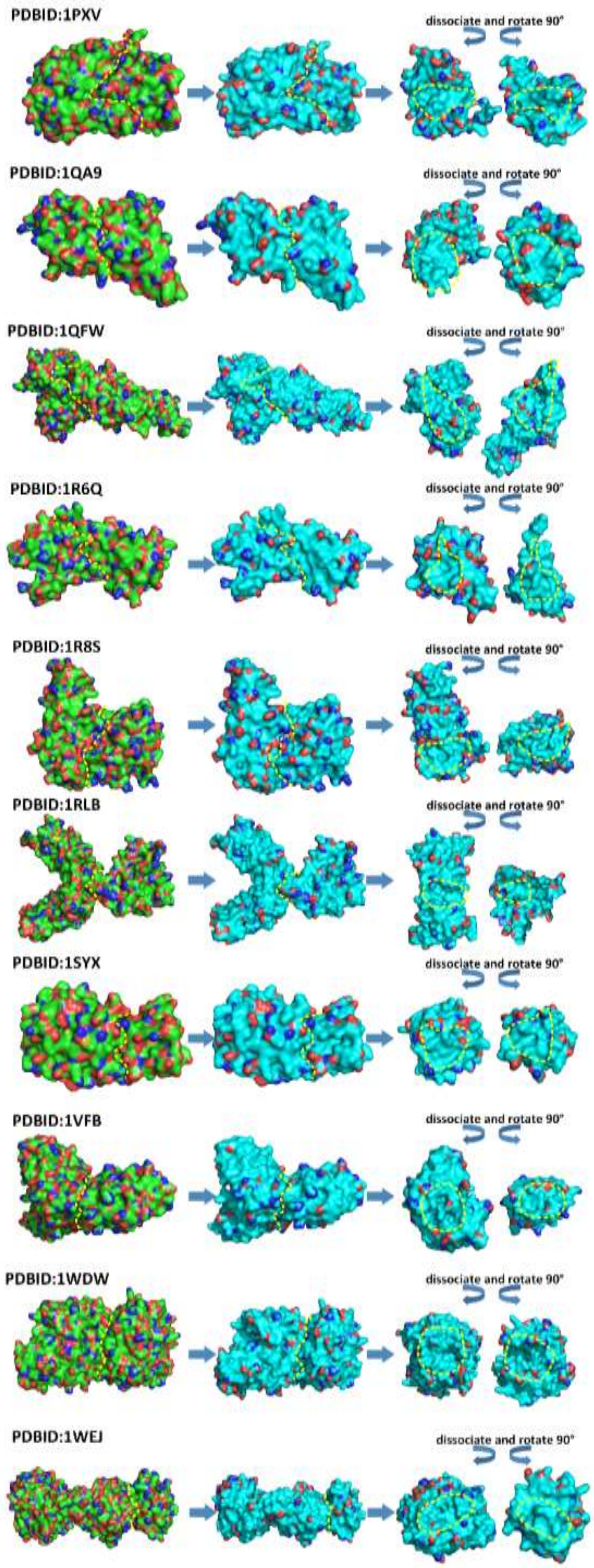
PDBID:1PXV
dissociate and rotate 90°
PDBID:1QA9
dissociate and rotate 90°
PDBID:1QFW
dissociate and rotate 90°
PDBID:1R6Q
dissociate and rotate 90°
PDBID:1R8S
dissociate and rotate 90°
PDBID:1RLB
dissociate and rotate 90°
PDBID:1SYX
dissociate and rotate 90°
PDBID:1VFB
dissociate and rotate 90°
PDBID:1WDW
dissociate and rotate 90°
PDBID:1WEJ
dissociate and rotate 90°

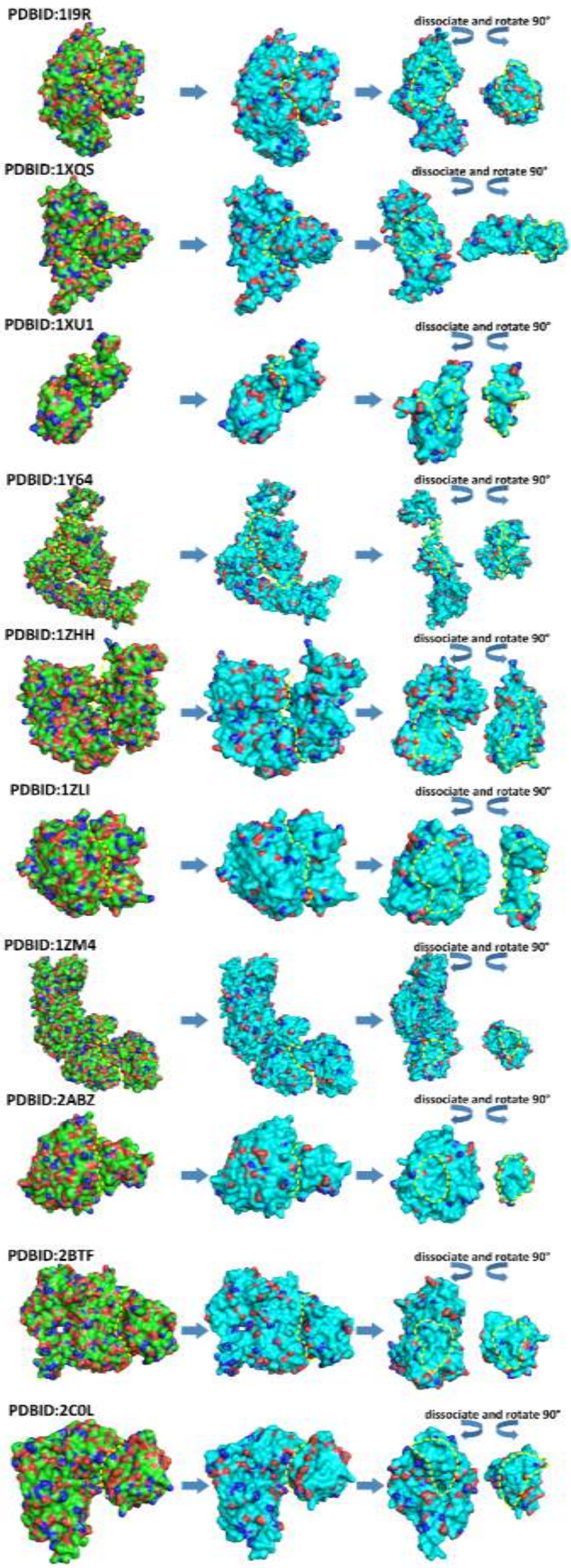
PDBID:1I9R
dissociate and rotate 90°
PDBID:1XQS
dissociate and rotate 90°
PDBID:1XU1
dissociate and rotate 90°
PDBID:1Y64
dissociate and rotate 90°
PDBID:1ZHH
dissociate and rotate 90°
PDBID:1ZLI
dissociate and rotate 90°
PDBID:1ZM4
dissociate and rotate 90°
PDBID:2ABZ
dissociate and rotate 90°
PDBID:2BTF
dissociate and rotate 90°
PDBID:2C0L
dissociate and rotate 90°

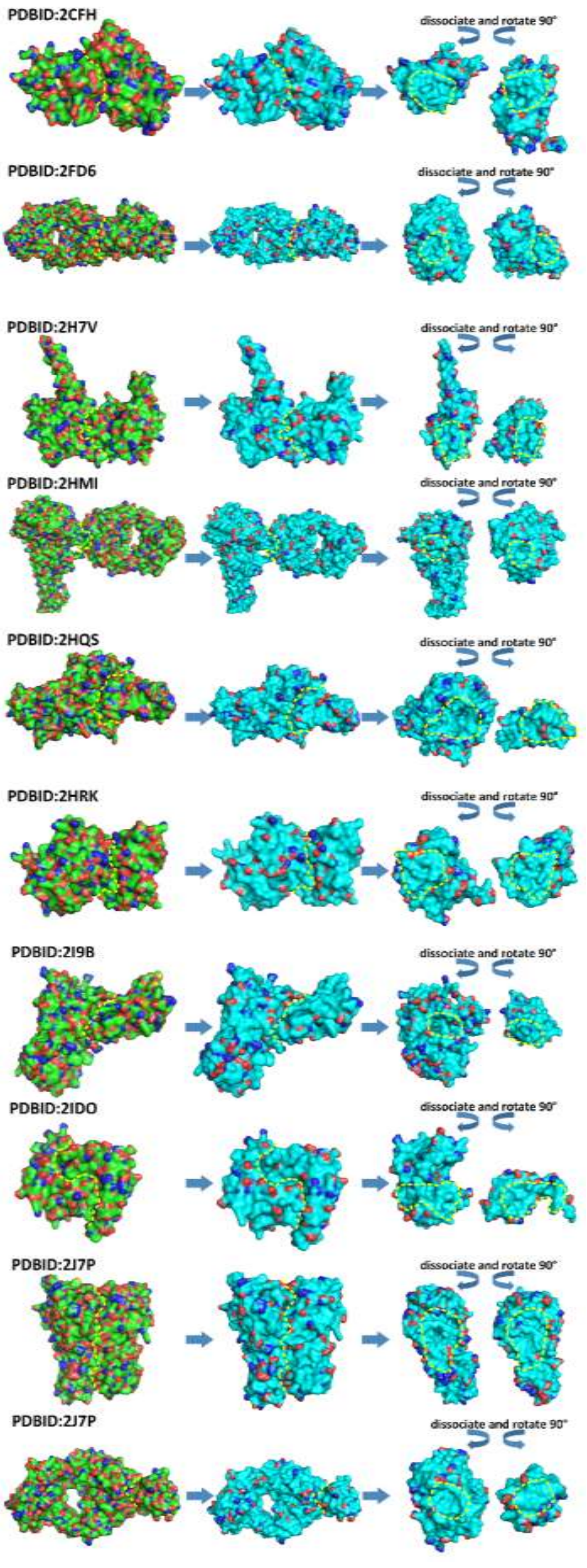
PDBID:2CFH
dissociate and rotate 90°
PDBID:2FD6
dissociate and rotate 90°
PDBID:2H7V
dissociate and rotate 90°
PDBID:2HMI
dissociate and rotate 90°
PDBID:2HQS
dissociate and rotate 90°
PDBID:2HRK
dissociate and rotate 90°
PDBID:2I9B
dissociate and rotate 90°
PDBID:2IDO
dissociate and rotate 90°
PDBID:2J7P
dissociate and rotate 90°
PDBID:2J7P
dissociate and rotate 90°

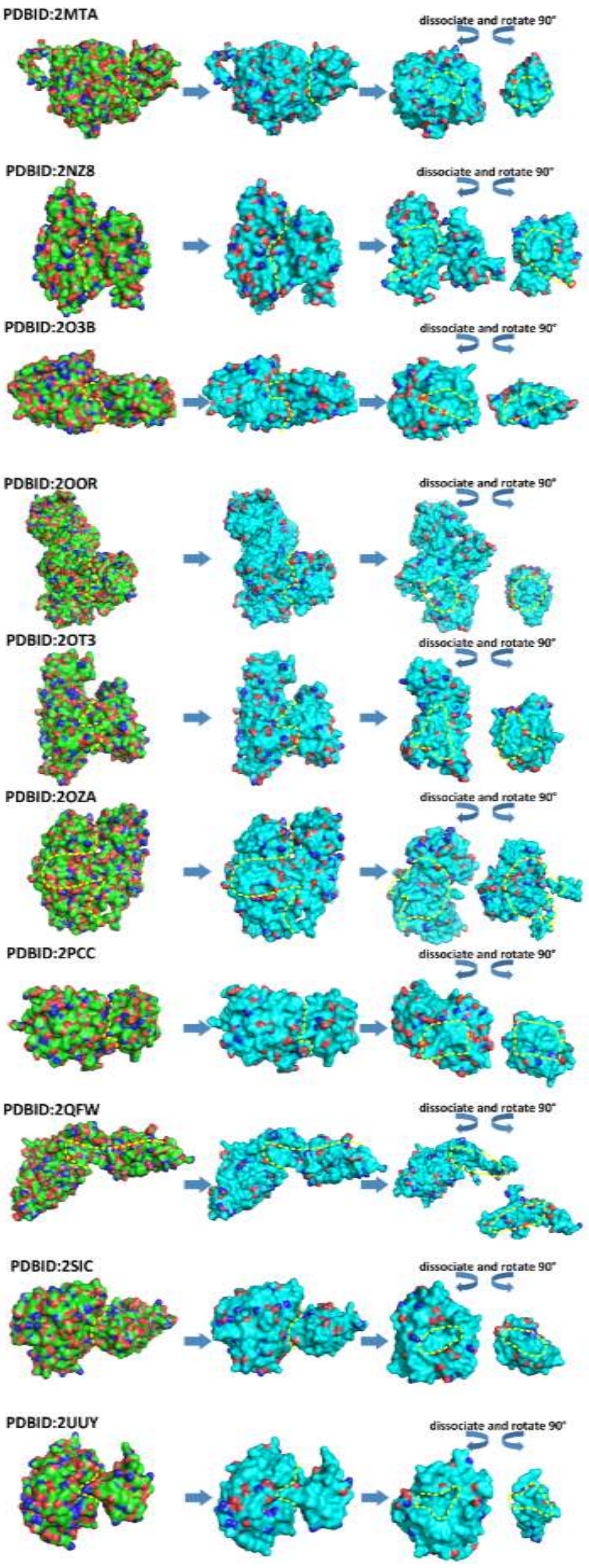
PDBID:2MTA
dissociate and rotate 90°
PDBID:2NZ8
dissociate and rotate 90°
PDBID:2O3B
dissociate and rotate 90°
PDBID:2OOR
dissociate and rotate 90°
PDBID:2OT3
dissociate and rotate 90°
PDBID:2OZA
dissociate and rotate 90°
PDBID:2PCC
dissociate and rotate 90°
PDBID:2QFW
dissociate and rotate 90°
PDBID:2SIC
dissociate and rotate 90°
PDBID:2UUY
dissociate and rotate 90°

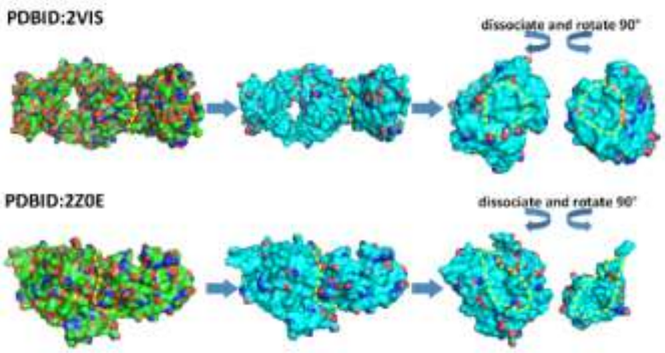

Fig.S3 Low-entropy regions of hydration shells covering the binding sites of proteins (from the protein–protein docking benchmark version 4.0 test system). The binding sites of the two proteins are highlighted by yellow dashed lines, and the low-entropy hydration shell region is highlighted in cyan. The remaining protein complex of the test system is illustrated in the Supplementary Information.

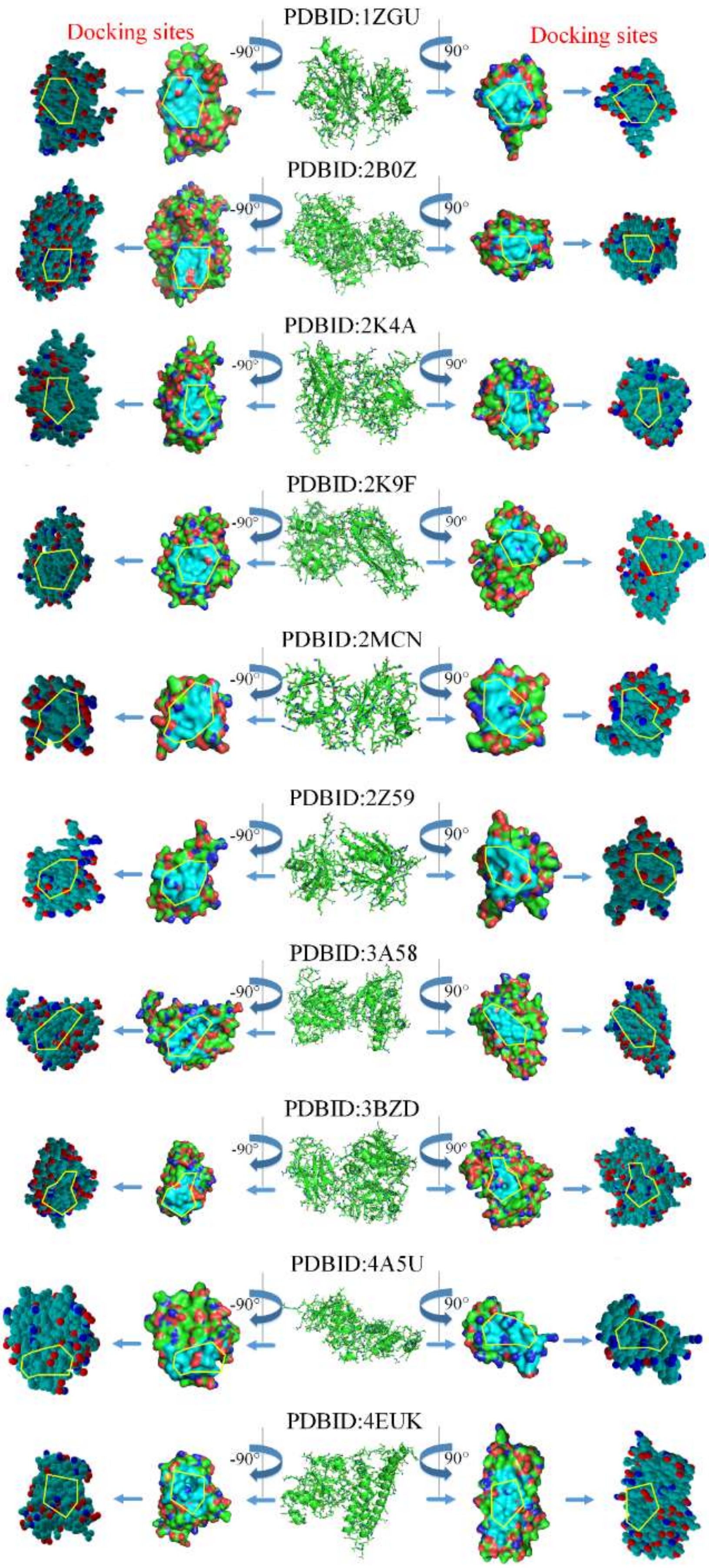

PDBID:1ZGU
Docking sites
-90°
90°
Docking sites
PDBID:2B0Z
-90°
90°
PDBID:2K4A
-90°
90°
PDBID:2K9F
-90°
90°
PDBID:2MCN
-90°
90°
PDBID:2Z59
-90°
90°
PDBID:3A58
-90°
90°
PDBID:3BZD
-90°
90°
PDBID:4A5U
-90°
90°
PDBID:4EUK
-90°
90°

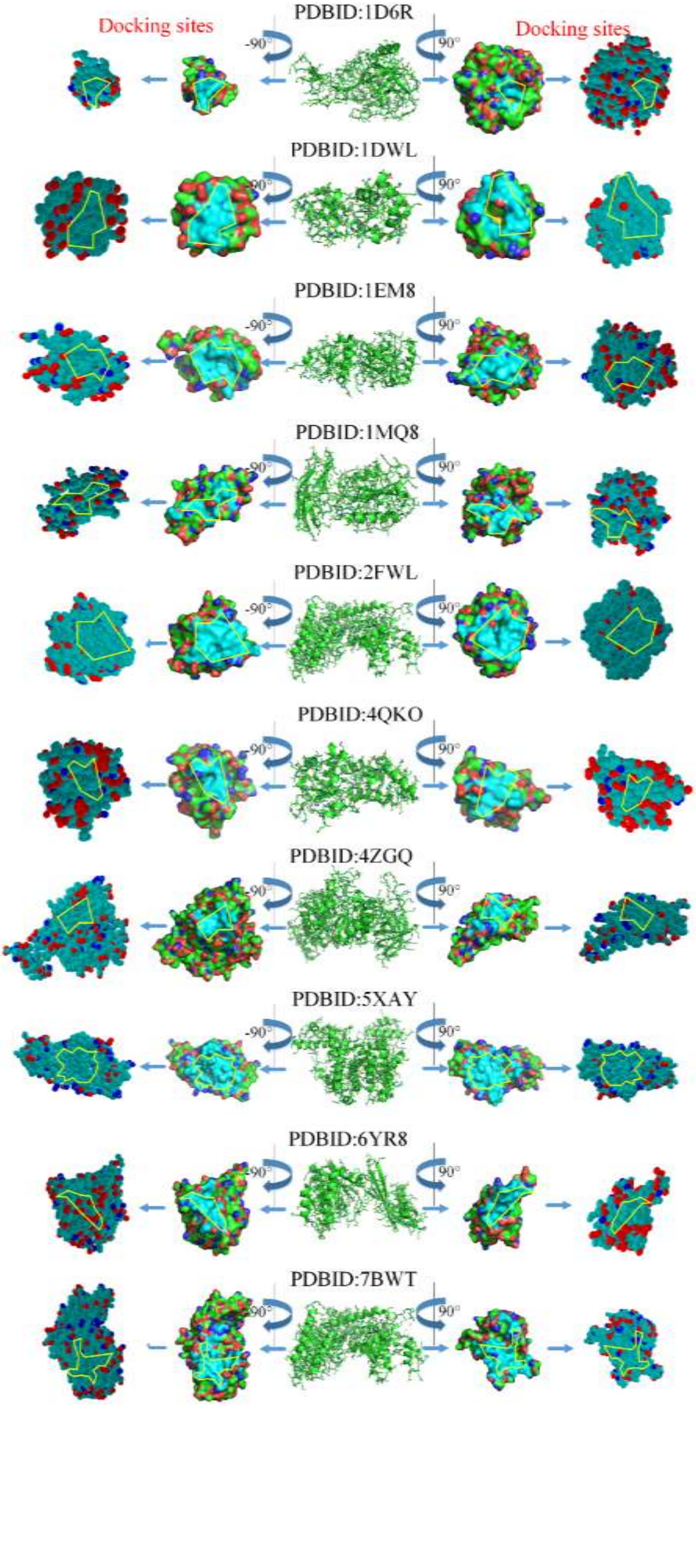

PDBID:1D6R
Docking sites
Docking sites
-90°
90°
PDBID:1DWL
PDBID:1EM8
PDBID:1MQ8
PDBID:2FWL
PDBID:4QKO
PDBID:4ZGQ
PDBID:5XAY
PDBID:6YR8
PDBID:7BWT

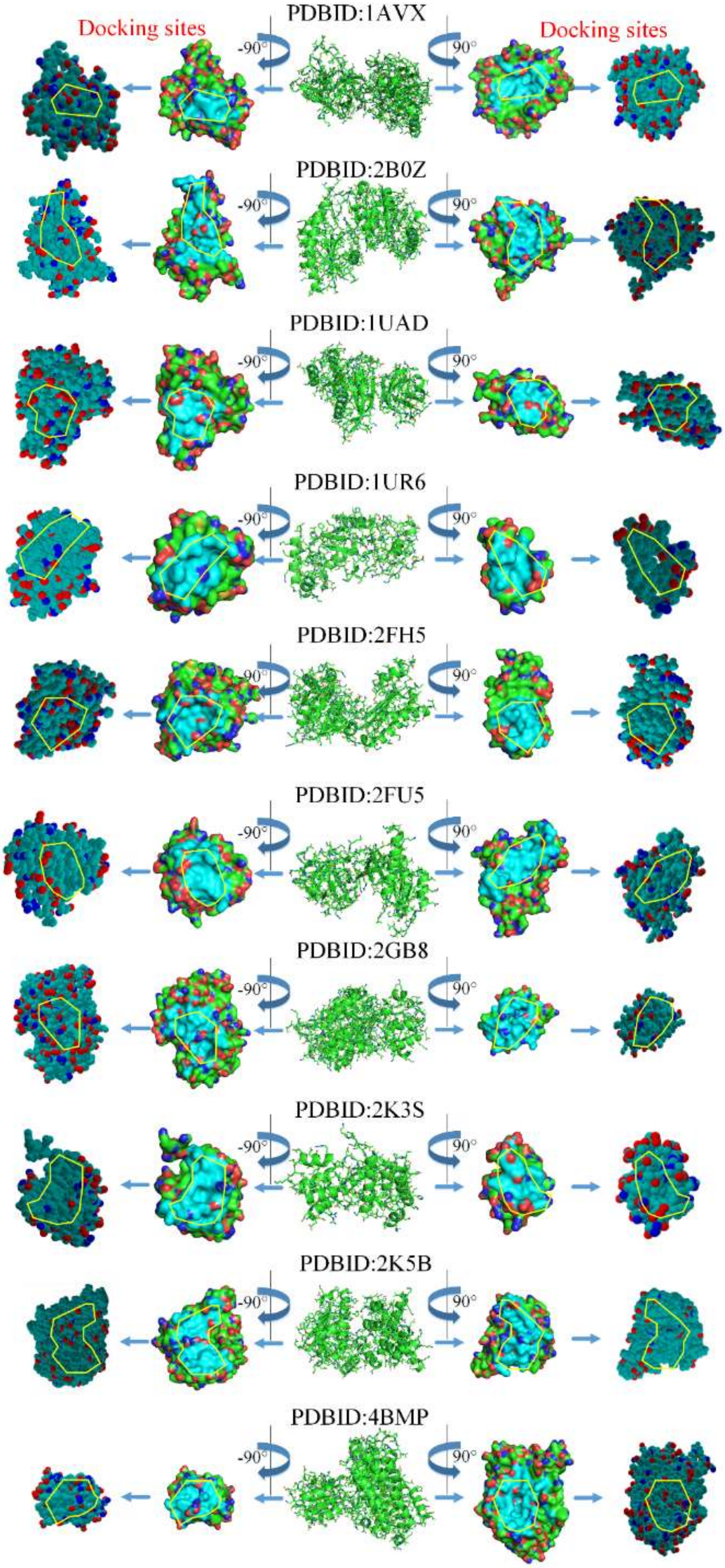

PDBID:1AVX
Docking sites
Docking sites
-90°
90°
PDBID:2B0Z
PDBID:1UAD
PDBID:1UR6
PDBID:2FH5
PDBID:2FU5
PDBID:2GB8
PDBID:2K3S
PDBID:2K5B
PDBID:4BMP

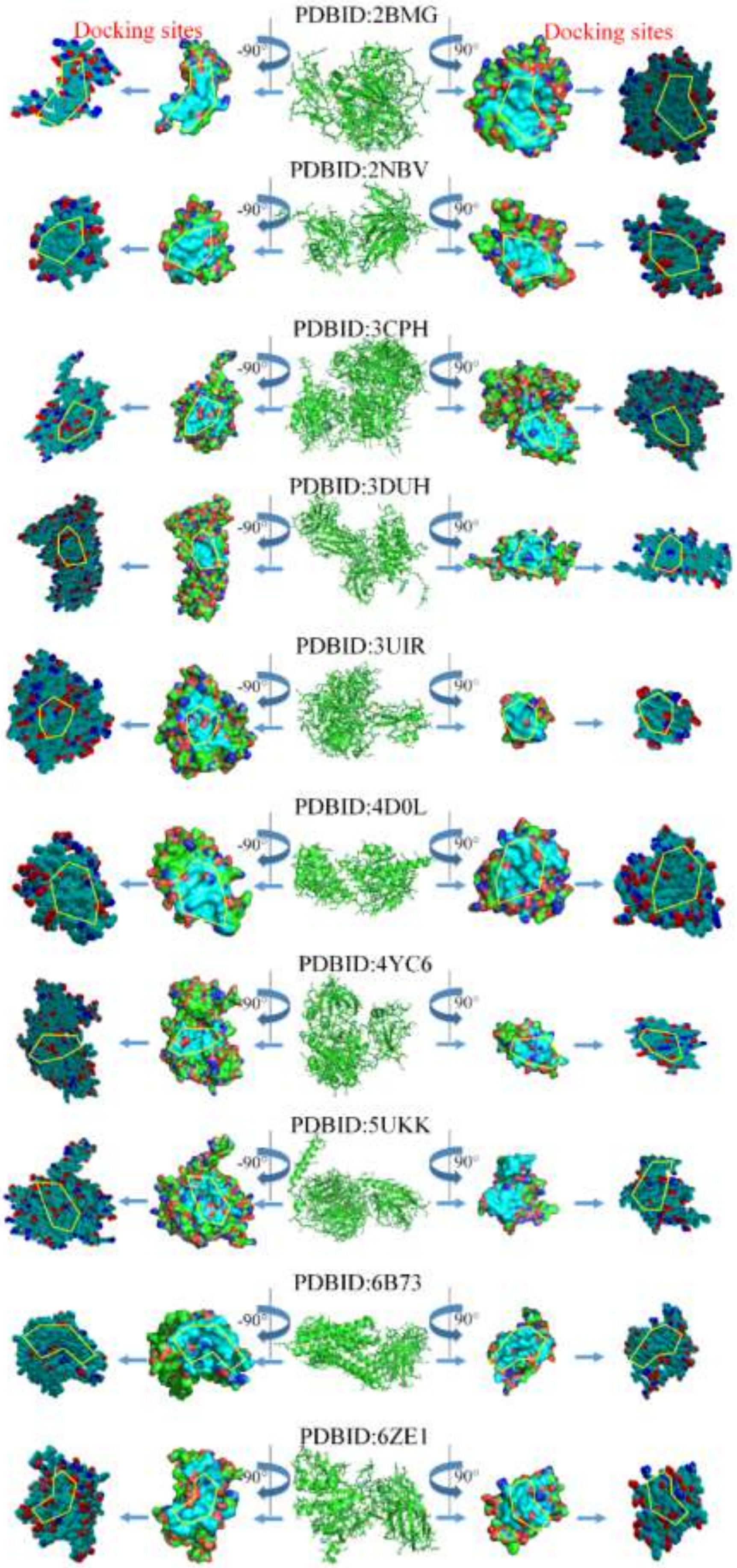

PDBID:2BMG
Docking sites
-90°
90°
Docking sites
PDBID:2NBV
PDBID:3CPH
PDBID:3DUH
PDBID:3UIR
PDBID:4D0L
PDBID:4YC6
PDBID:5UKK
PDBID:6B73
PDBID:6ZE1

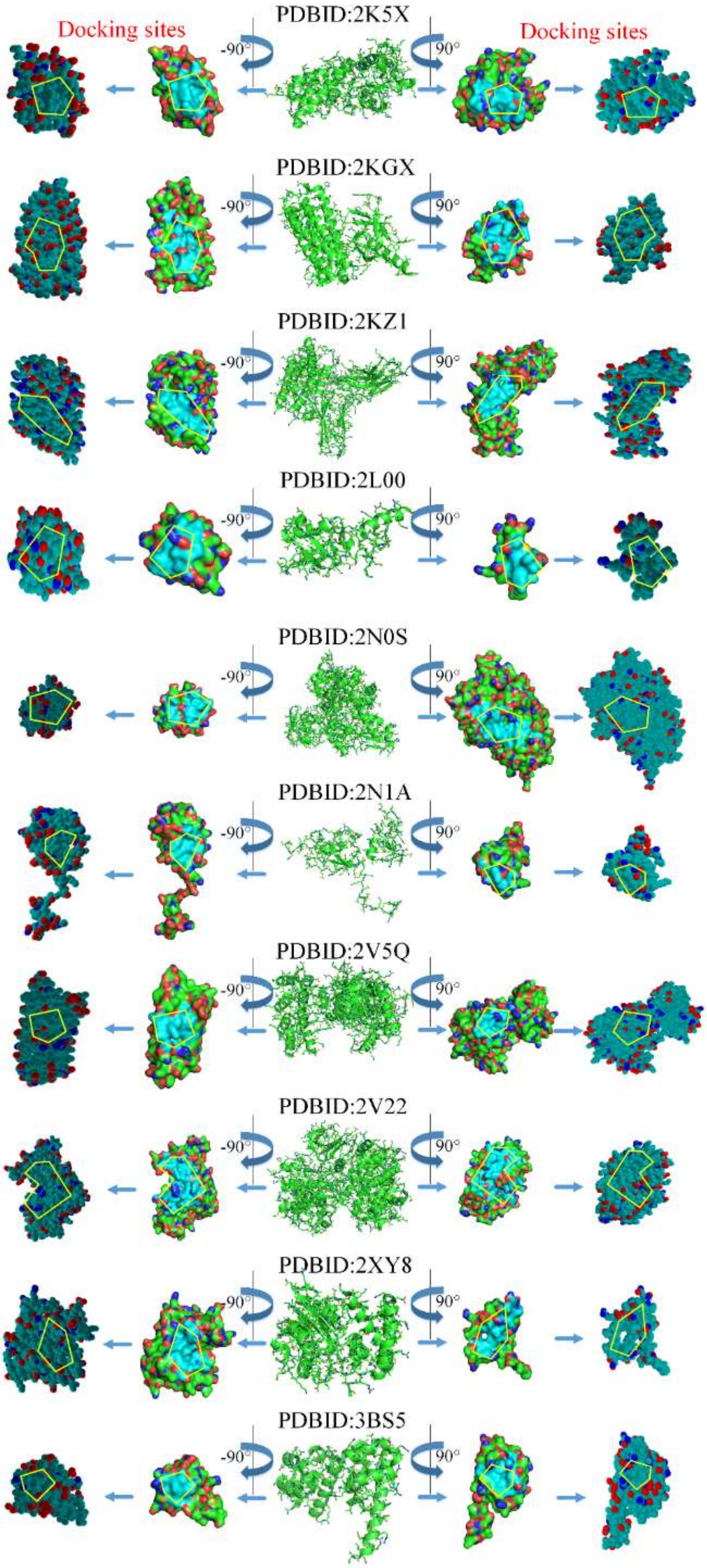
PDBID:2K5X
Docking sites
Docking sites
-90°
90°
PDBID:2KGX
PDBID:2KZ1
PDBID:2L00
PDBID:2N0S
PDBID:2N1A
PDBID:2V5Q
PDBID:2V22
PDBID:2XY8
PDBID:3BS5

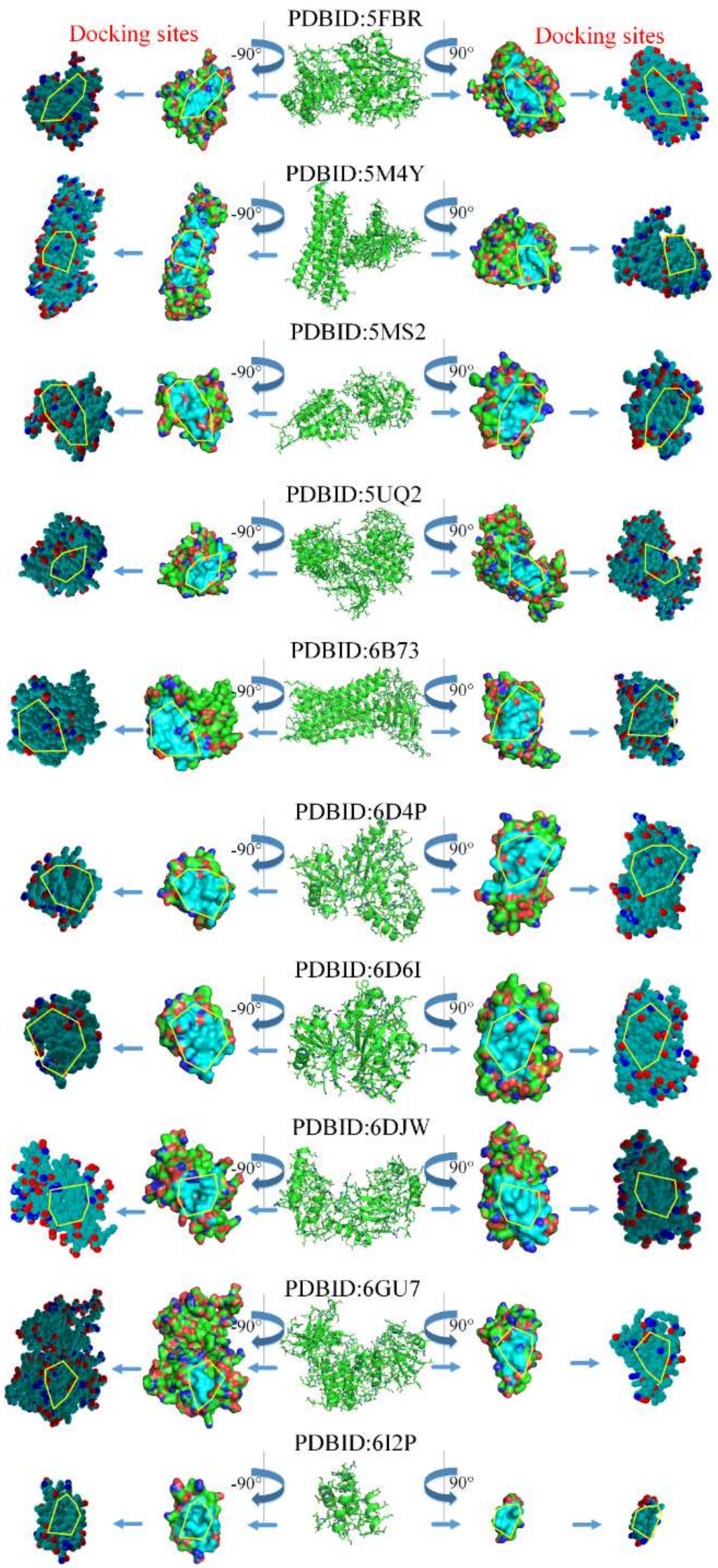

Docking sites
Docking sites
PDBID:5FBR
PDBID:5M4Y
PDBID:5MS2
PDBID:5UQ2
PDBID:6B73
PDBID:6D4P
PDBID:6D6I
PDBID:6DJW
PDBID:6GU7
PDBID:6I2P
-90°
90°

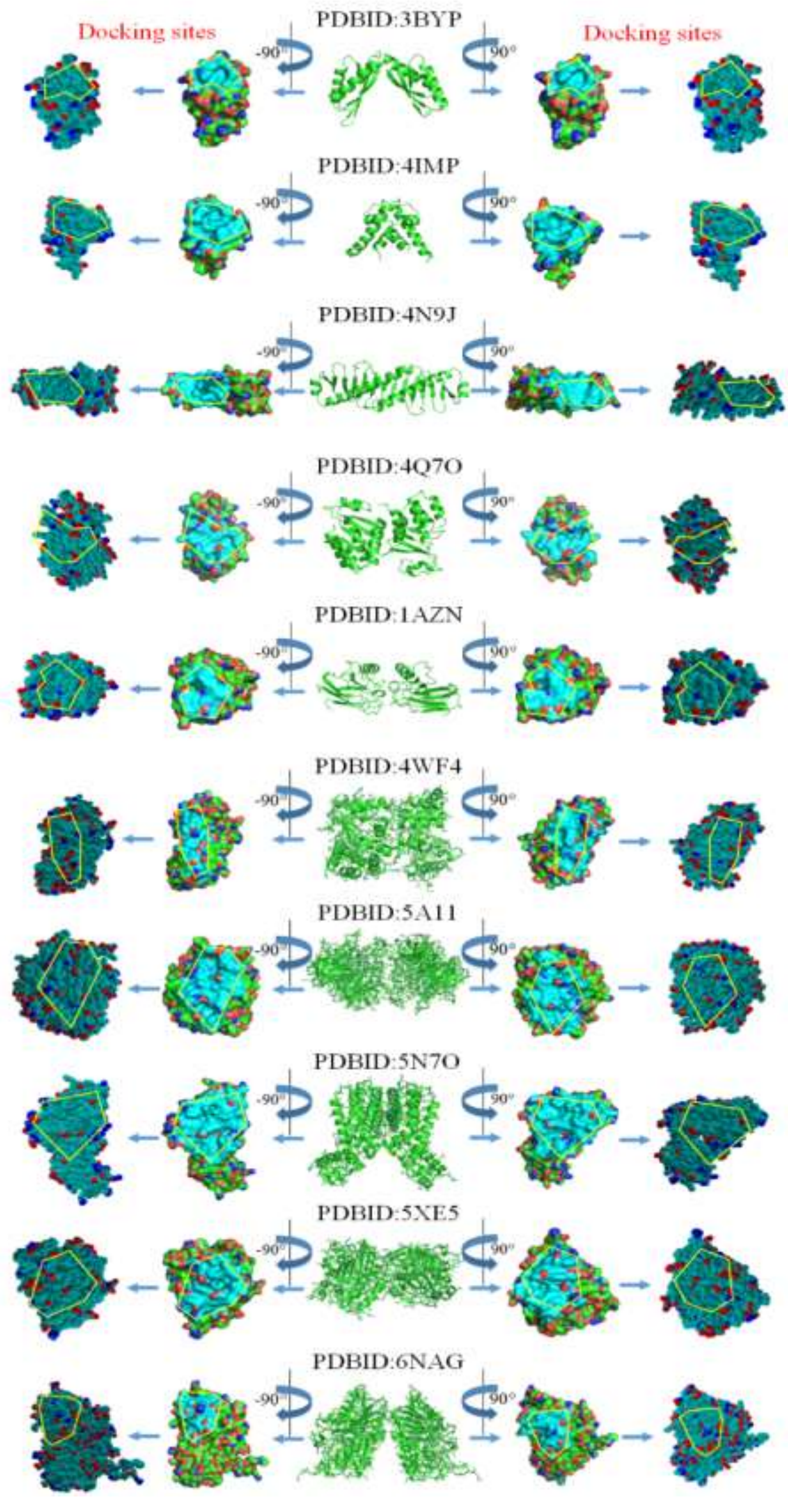

Docking sites
Docking sites
PDBID:3BYP
-90°
90°
PDBID:4IMP
-90°
90°
PDBID:4N9J
-90°
90°
PDBID:4Q7O
-90°
90°
PDBID:1AZN
-90°
90°
PDBID:4WF4
-90°
90°
PDBID:5A11
-90°
90°
PDBID:5N7O
-90°
90°
PDBID:5XE5
-90°
90°
PDBID:6NAG
-90°
90°

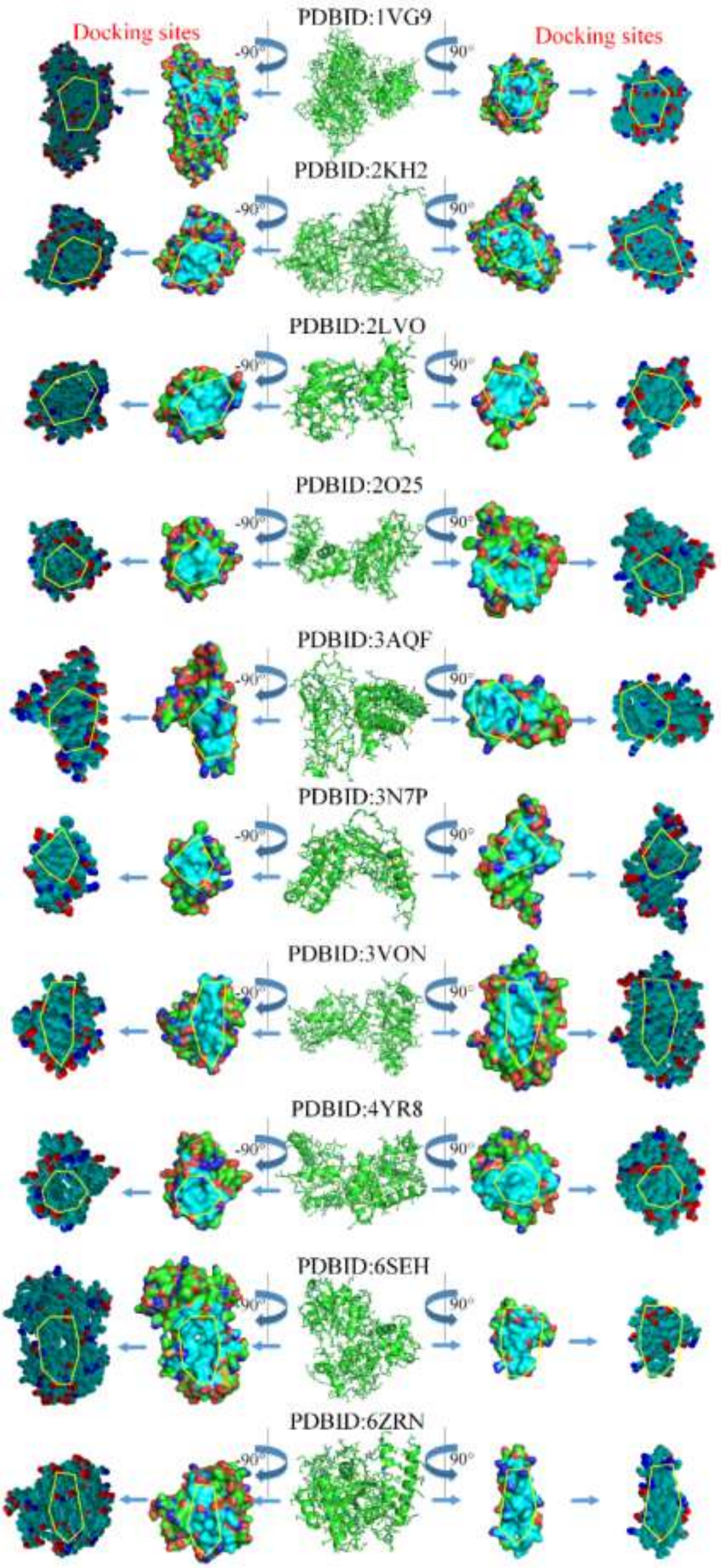

PDBID:1VG9
Docking sites
-90°
90°
Docking sites
PDBID:2KH2
PDBID:2LVO
PDBID:2O25
PDBID:3AQF
PDBID:3N7P
PDBID:3VON
PDBID:4YR8
PDBID:6SEH
PDBID:6ZRN

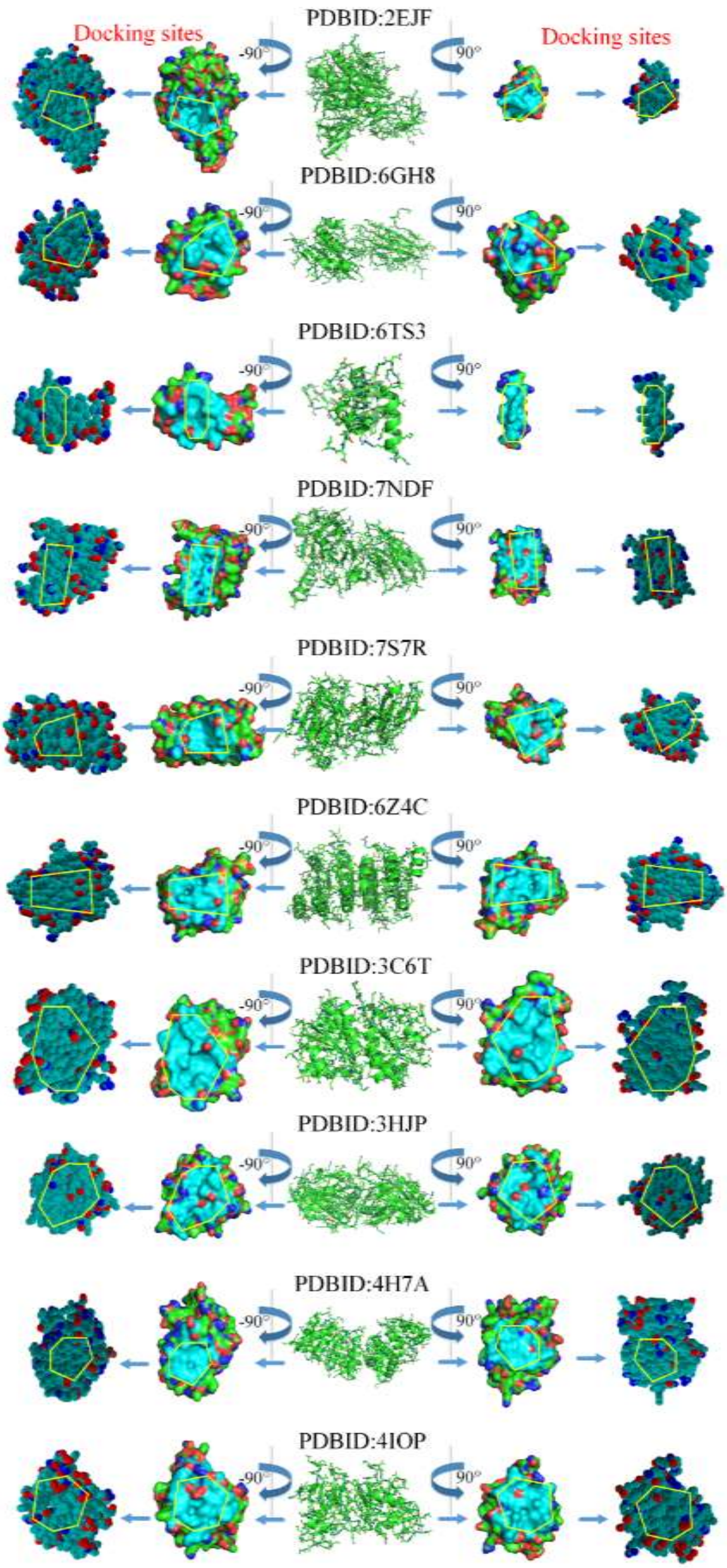
PDBID:2EJF
Docking sites
-90°
90°
Docking sites
PDBID:6GH8
-90°
90°
PDBID:6TS3
-90°
90°
PDBID:7NDF
-90°
90°
PDBID:7S7R
-90°
90°
PDBID:6Z4C
-90°
90°
PDBID:3C6T
-90°
90°
PDBID:3HJP
-90°
90°
PDBID:4H7A
-90°
90°
PDBID:4IOP
-90°
90°

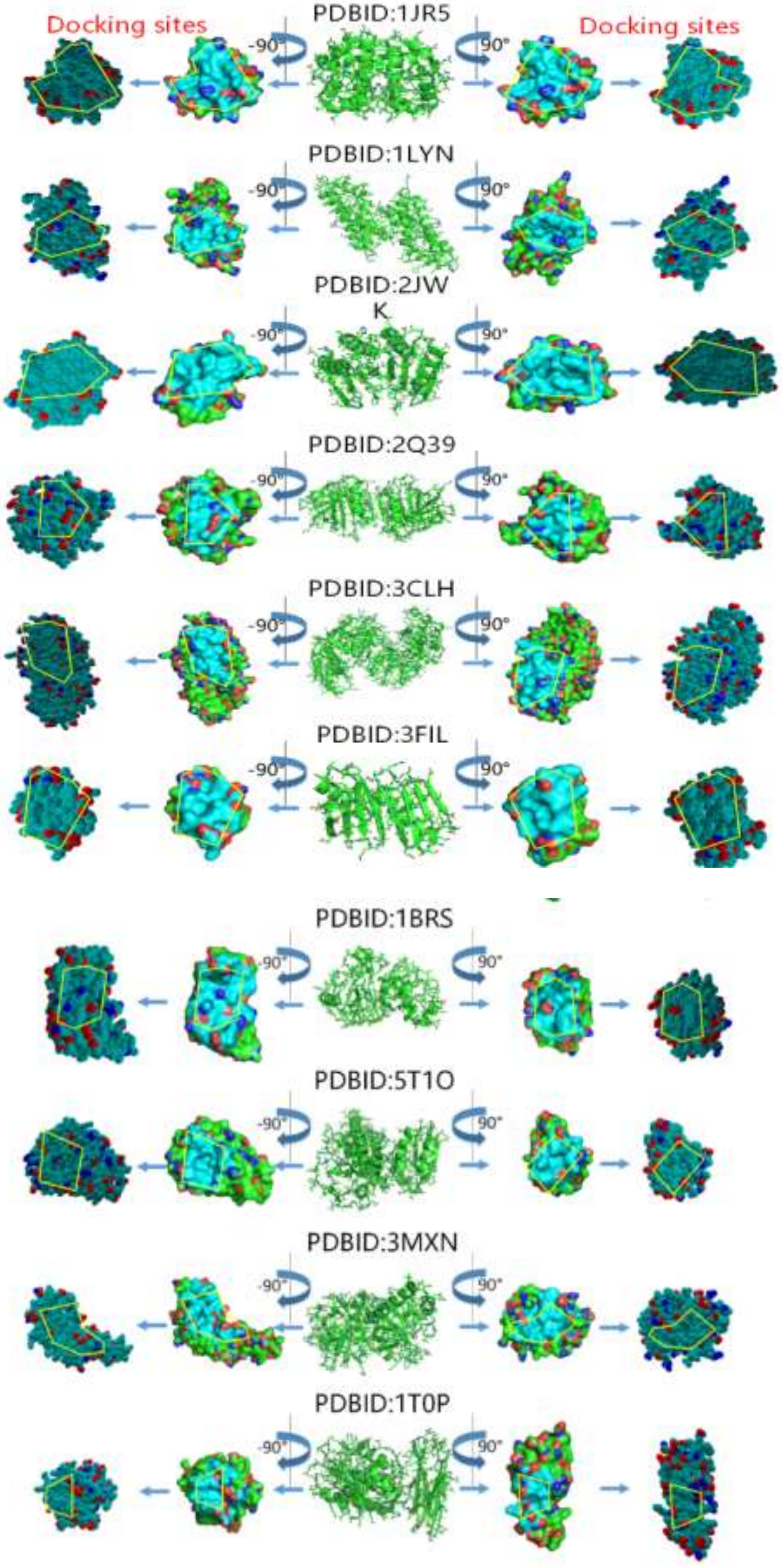
Docking sites
PDBID:1JR5
Docking sites
-90°
90°
PDBID:1LYN
PDBID:2JW
K
PDBID:2Q39
PDBID:3CLH
PDBID:3FIL
PDBID:1BRS
PDBID:5T1O
PDBID:3MXN
PDBID:1T0P

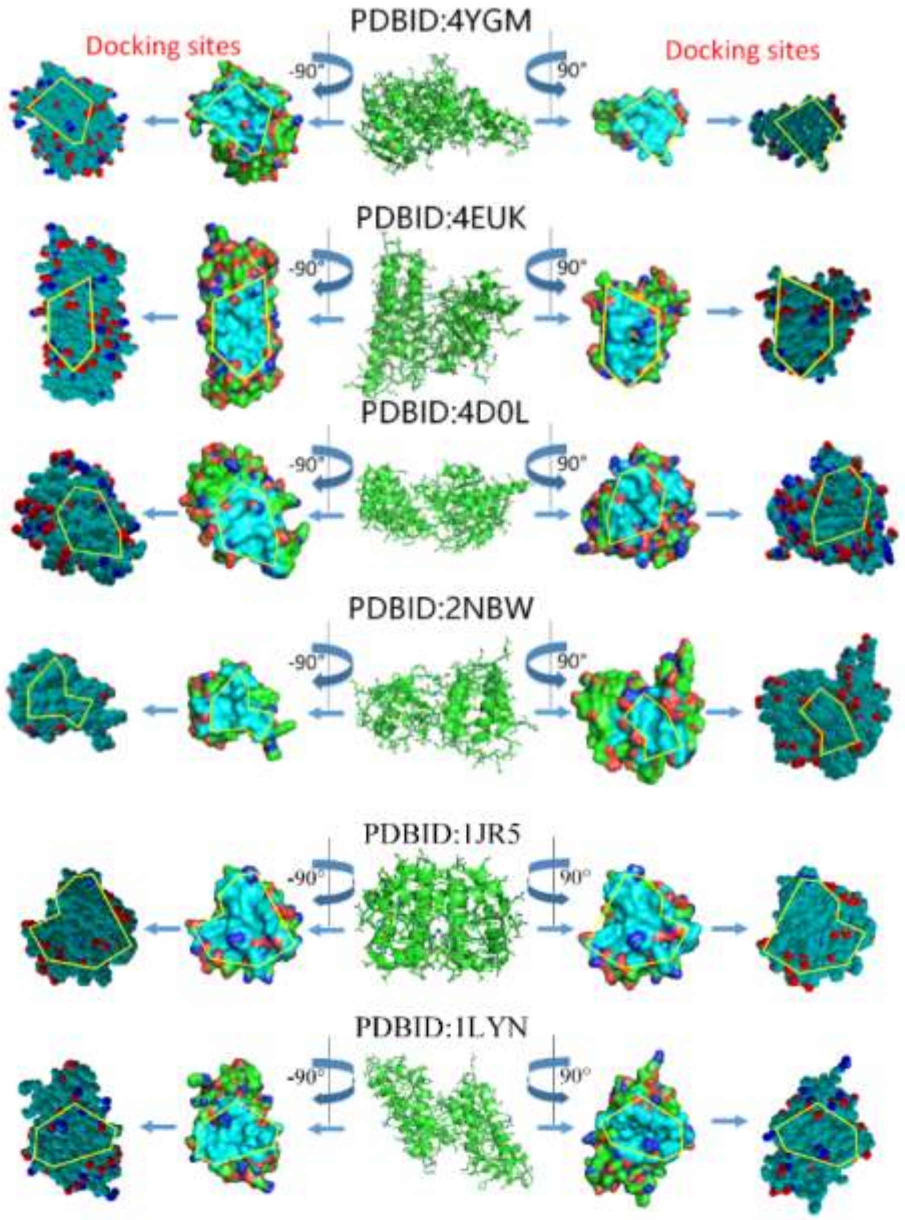

Fig.S4 The prediction of binding sites of 106 protein complexes, which randomly select from Protein Data Bank, through identifying shape-matched the largest low-entropy hydration shells of individual protein.

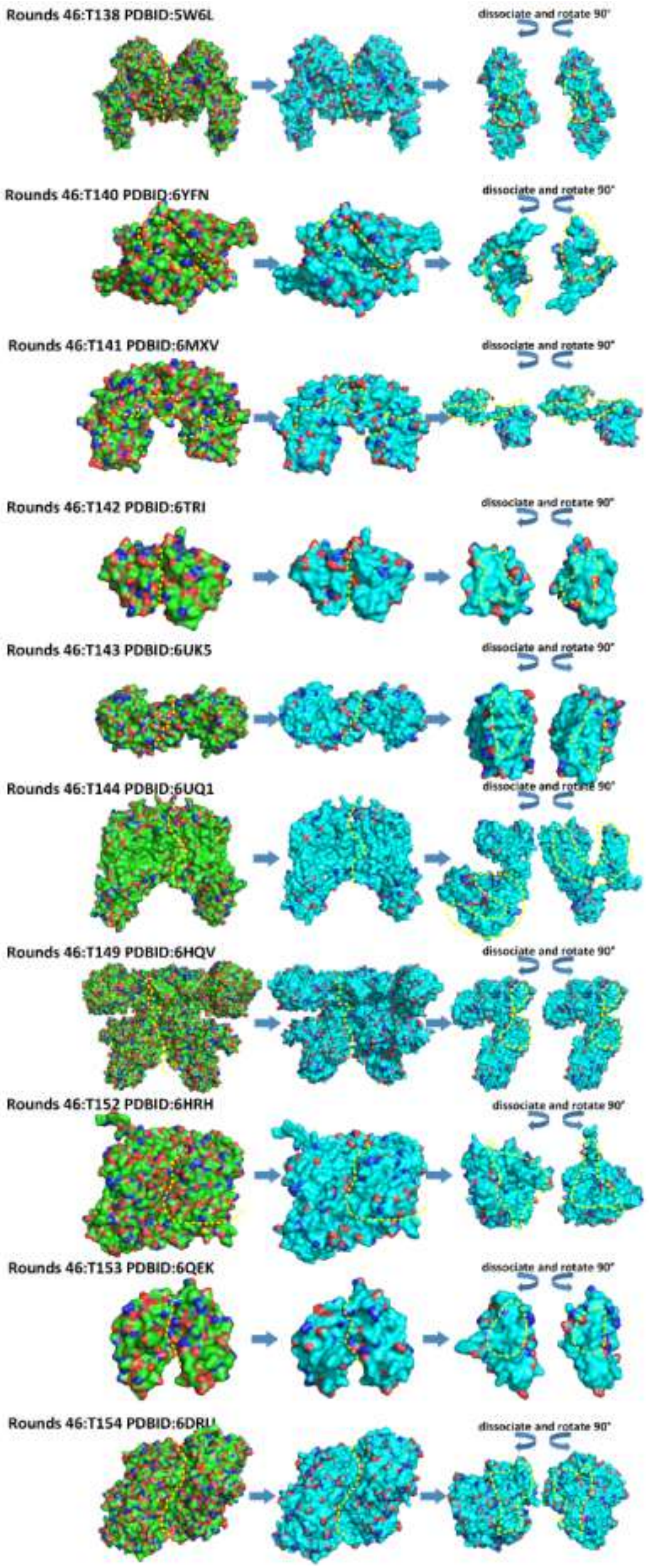
Rounds 46:T138 PDBID:5W6L
dissociate and rotate 90°
Rounds 46:T140 PDBID:6YFN
dissociate and rotate 90°
Rounds 46:T141 PDBID:6MXV
dissociate and rotate 90°
Rounds 46:T142 PDBID:6TRI
dissociate and rotate 90°
Rounds 46:T143 PDBID:6UK5
dissociate and rotate 90°
Rounds 46:T144 PDBID:6UQ1
dissociate and rotate 90°
Rounds 46:T149 PDBID:6HQV
dissociate and rotate 90°
Rounds 46:T152 PDBID:6HRH
dissociate and rotate 90°
Rounds 46:T153 PDBID:6QEK
dissociate and rotate 90°
Rounds 46:T154 PDBID:6DRU
dissociate and rotate 90°

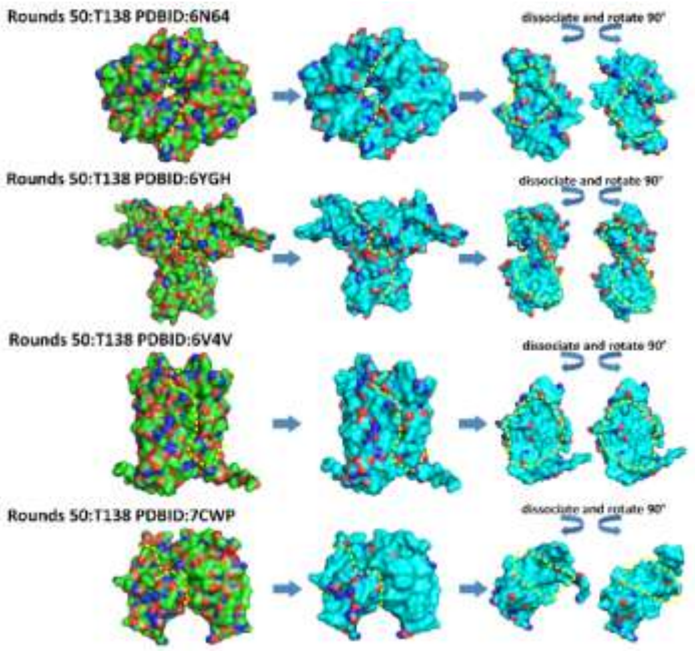

Fig.S5 The prediction of binding sites of protein pairs from CAPRI rounds 46 and 50 test systems by identifying the shape-matched low-entropy regions of hydration shells of individual proteins.

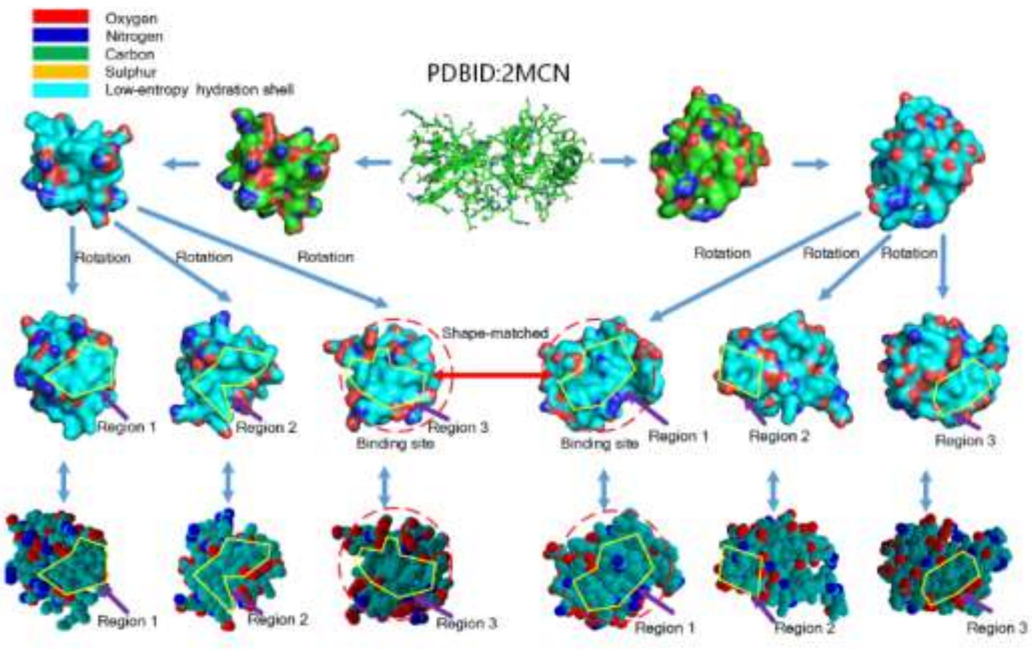

Fig. S6 The prediction of binding sites of a protein pair through identifying the shape-matched low-entropy regions of hydration shells of individual proteins.

Supplementary Table S1. Summary of the proportion of genuine hydrophilic atoms area to hydrophobic atoms area in Dockground test system. P_{Ao} and P_{Ai} represent respectively proportion of genuine hydrophilic atoms areas at the outside the binding site regions and the inside the binding site regions of one protein subunit of the complex. P_{Bo} and P_{Bi} represent respectively proportion of genuine hydrophilic atoms areas at the outside the binding site regions and inside the binding site region of the other protein subunit of the complex.

PDBID	P_{Ao}	P_{Ai}	P_{Bo}	P_{Bi}
1AKJ	0.15	0.06	0.13	0.07
1AY7	0.16	0.07	0.17	0.04
1B27	0.11	0.06	0.21	0.05
1BDJ	0.14	0.07	0.15	0.13
1BGX	0.16	0.04	0.12	0.03
1BUH	0.11	0.05	0.22	0.06
1BUI	0.13	0.06	0.18	0.05
1BVN	0.13	0.04	0.22	0.06
1CC0	0.18	0.04	0.15	0.01
1CLV	0.14	0.04	0.68	0.04
1DE4	0.11	0.06	0.15	0.07
1DFJ	0.16	0.07	0.21	0.07
1E6J	0.09	0.01	0.14	0.04
1EAY	0.15	0.01	0.15	0.07
1EER	0.15	0.08	0.16	0.04
1EWY	0.14	0.04	0.20	0.07
1F6M	0.13	0.02	0.19	0.03
1F80	0.13	0.07	0.23	0.16
1F93	0.17	0.08	0.14	0.10
1FCC	0.15	0.06	0.25	0.06
1FLE	0.15	0.03	0.16	0.05
1FLT	0.20	0.04	0.15	0.05
1FQ1	0.12	0.03	0.14	0.07
1FRT	0.15	0.05	0.16	0.03
1G4B	0.16	0.03	0.13	0.09
1G6V	0.11	0.03	0.12	0.07
1G73	0.17	0.04	0.14	0.04
1GCQ	0.14	0.05	0.18	0.10
1GG2	0.14	0.05	0.15	0.06
1GLB	0.12	0.06	0.15	0.05
1GPQ	0.13	0.06	0.15	0.07
1GPW	0.15	0.03	0.17	0.05
1HE1	0.12	0.05	0.19	0.02
1HIA	0.17	0.02	0.13	0.09
1HJA	0.12	0.01	0.19	0.04
1HYR	0.18	0.07	0.16	0.03

1I2M	0.13	0.08	0.12	0.06
1I4E	0.13	0.07	0.16	0.06
1I8L	0.15	0.05	0.14	0.05
1IB1	0.18	0.06	0.14	0.13
1ICF	0.15	0.04	0.25	0.05
1IM9	0.16	0.08	0.14	0.04
1IS7	0.13	0.04	0.15	0.07
1JIW	0.12	0.04	0.18	0.03
1JK9	0.18	0.04	0.15	0.04
1JTD	0.09	0.07	0.12	0.05
1JZD	0.12	0.03	0.26	0.03
1K5G	0.17	0.06	0.14	0.06
1KG0	0.14	0.05	0.11	0.09
1KGY	0.14	0.04	0.17	0.03
1KYO	0.11	0.03	0.16	0.04
1L9J	0.07	0.05	0.18	0.03
1LB2	0.14	0.07	0.17	0.08
1LFD	0.17	0.06	0.23	0.06
1LTX	0.12	0.05	0.13	0.03
1LX5	0.13	0.03	0.23	0.04
1M63	0.15	0.04	0.14	0.05
1MG2	0.11	0.02	0.12	0.04
1ML0	0.11	0.03	0.30	0.06
1MQ8	0.18	0.03	0.14	0.05
1N8O	0.11	0.01	0.15	0.02
1NB5	0.14	0.02	0.21	0.02
1NBF	0.17	0.06	0.29	0.07
1NQL	0.15	0.04	0.58	0.04
1NW9	0.11	0.03	0.18	0.04
1OC0	0.12	0.04	0.23	0.06
1OFU	0.13	0.03	0.13	0.05
1P8V	0.13	0.05	0.13	0.07
1PVH	0.15	0.03	0.13	0.10
1QAV	0.14	0.04	0.15	0.04
1R5I	0.13	0.04	0.15	0.09
1R8S	0.20	0.07	0.18	0.07
1RPQ	0.18	0.04	0.51	0.06
1S1Q	0.13	0.04	0.21	0.07
1S78	0.15	0.03	0.10	0.04
1SJH	0.13	0.04	0.13	0.08
1TCO	0.13	0.05	0.15	0.06
1TE1	0.11	0.03	0.15	0.05
1TMQ	0.14	0.04	0.17	0.02
1TZN	0.14	0.05	0.13	0.05

1U0N	0.12	0.08	0.14	0.05
1UAD	0.17	0.04	0.15	0.03
1UUG	0.13	0.05	0.33	0.06
1V5I	0.09	0.03	0.21	0.09
1V7P	0.14	0.01	0.11	0.05
1VFB	0.10	0.03	0.14	0.07
1VG0	0.13	0.06	0.17	0.05
1VRS	0.22	0.04	0.19	0.04
1W1I	0.11	0.04	0.13	0.09
1WEJ	0.10	0.04	0.13	0.07
1WQ1	0.17	0.04	0.19	0.09
1WRD	0.18	0.05	0.20	0.05
1X86	0.18	0.05	0.17	0.07
1XK4	0.13	0.05	0.30	0.07
1XT9	0.16	0.04	0.24	0.08
1Y64	0.16	0.06	0.14	0.05
1Y8R	0.13	0.04	0.22	0.14
1Y8X	0.16	0.06	0.19	0.05
1YCS	0.16	0.07	0.13	0.08
1YU6	0.09	0.01	0.23	0.02
1YVB	0.16	0.03	0.18	0.02
1Z3G	0.10	0.03	0.18	0.07
1Z5Y	0.17	0.02	0.20	0.02
1ZLH	0.10	0.04	0.21	0.04
1ZUD	0.13	0.04	0.26	0.12
2A1T	0.11	0.05	0.11	0.05
2A41	0.12	0.04	0.12	0.04
2A5D	0.16	0.05	0.13	0.07
2A9K	0.10	0.06	0.19	0.06
2ABZ	0.10	0.05	0.14	0.05
2AQ3	0.16	0.04	0.15	0.04
2B4S	0.13	0.04	0.13	0.04
2BCG	0.12	0.03	0.16	0.05
2BCJ	0.14	0.05	0.16	0.06
2BCN	0.13	0.04	0.15	0.06
2BKU	0.16	0.05	0.19	0.05
2BOV	0.13	0.02	0.17	0.04
2BQ1	0.13	0.04	0.13	0.07
2BWE	0.19	0.04	0.30	0.05
2C0L	0.16	0.04	0.16	0.05
2C2V	0.15	0.04	0.16	0.04
2CH4	0.17	0.03	0.22	0.03
2D5R	0.14	0.03	0.19	0.03
2DD8	0.09	0.02	0.11	0.04

2EJF	0.12	0.02	0.20	0.10
2G45	0.19	0.04	0.16	0.08
2GJ7	0.13	0.04	0.16	0.05
2GRX	0.13	0.04	0.37	0.10
2GWF	0.13	0.07	0.20	0.05
2H1L	0.12	0.03	0.13	0.10
2HJ9	0.14	0.05	0.14	0.04
2HQS	0.12	0.04	0.18	0.07
2HRK	0.12	0.04	0.15	0.04
2I25	0.15	0.06	0.12	0.08
2IDO	0.15	0.04	0.31	0.03
2IJO	0.15	0.03	0.12	0.05
2IWT	0.13	0.05	0.15	0.03
2J12	0.09	0.05	0.14	0.06
2NQD	0.15	0.04	0.16	0.01
2NVY	0.13	0.04	0.18	0.07
2NXN	0.15	0.06	0.22	0.03
2O25	0.13	0.01	0.16	0.01
2O2V	0.15	0.09	0.19	0.05
2O3B	0.12	0.05	0.15	0.10
2O8V	0.16	0.05	0.16	0.04
2OOB	0.20	0.05	0.23	0.03
2PU9	0.17	0.05	0.17	0.01
2QYI	0.10	0.02	0.18	0.06
2RII	0.12	0.01	0.39	0.03
2SGE	0.11	0.01	0.16	0.08
2UY7	0.17	0.06	0.21	0.07
2V55	0.15	0.06	0.15	0.08
2VDB	0.12	0.04	0.26	0.03
2VRR	0.12	0.01	0.22	0.03
2WBW	0.11	0.03	0.16	0.03
2WY7	0.12	0.04	0.23	0.09
2WY8	0.11	0.08	0.24	0.07
2X0B	0.16	0.05	0.14	0.02
2X9A	0.16	0.02	0.27	0.06
2XGY	0.10	0.04	0.13	0.07
2XN9	0.13	0.06	0.13	0.07
2XWB	0.14	0.06	0.15	0.10
2XZ1	0.12	0.08	0.21	0.10
2YVJ	0.12	0.04	0.17	0.04
2ZAE	0.16	0.06	0.19	0.07
2ZU0	0.14	0.06	0.17	0.04
2ZVN	0.26	0.05	0.22	0.05
3A1P	0.21	0.06	0.18	0.06

3A8I	0.12	0.05	0.17	0.07
3AV0	0.17	0.05	0.17	0.08
3BH6	0.15	0.06	0.16	0.07
3BK3	0.16	0.02	0.26	0.02
3BP8	0.14	0.05	0.14	0.04
3BS5	0.17	0.12	0.18	0.12
3BSZ	0.13	0.02	0.16	0.04
3BUZ	0.16	0.01	0.13	0.04
3BX1	0.12	0.01	0.16	0.03
3BX7	0.12	0.03	0.18	0.04
3CBK	0.15	0.05	0.17	0.05
3CII	0.18	0.11	0.18	0.08
3CU1	0.18	0.04	0.15	0.03
3CVH	0.09	0.06	0.14	0.07
3CW2	0.14	0.04	0.20	0.05
3D3C	0.14	0.08	0.24	0.08
3D5O	0.12	0.05	0.15	0.05
3D5R	0.11	0.08	0.22	0.05
3DAW	0.13	0.03	0.19	0.06
3E2L	0.09	0.02	0.15	0.03
3F1P	0.19	0.05	0.16	0.04
3F7P	0.15	0.08	0.17	0.08
3FAP	0.15	0.03	0.15	0.06
3FDS	0.14	0.04	0.14	0.02
3FN1	0.15	0.05	0.22	0.04
3FPU	0.28	0.06	0.26	0.07
3G3A	0.13	0.07	0.17	0.08
3G6D	0.11	0.01	0.20	0.03
3GE3	0.11	0.04	0.37	0.10
3HI6	0.10	0.02	0.11	0.06
3HZI	0.12	0.01	0.15	0.11
3K1I	0.21	0.03	0.21	0.07
3K9M	0.15	0.03	0.24	0.05
3KLD	0.13	0.04	0.13	0.07
3L1Z	0.17	0.01	0.24	0.08
3L4Q	0.22	0.06	0.17	0.04
3L5N	0.15	0.07	0.24	0.05
3L89	0.12	0.06	0.19	0.02
3LB8	0.13	0.03	0.20	0.06
3LTF	0.15	0.06	0.63	0.07
3LVJ	0.12	0.06	0.15	0.07
3LVL	0.12	0.05	0.20	0.01
3LWN	0.15	0.05	0.17	0.07
3M18	0.17	0.03	0.14	0.08

3MJ7	0.16	0.03	0.14	0.04
3MJ9	0.10	0.04	0.18	0.06
3MXW	0.09	0.07	0.15	0.08
3MZW	0.15	0.05	0.24	0.10
3OED	0.13	0.06	0.11	0.04
3OJ4	0.14	0.02	0.28	0.06
3ONA	0.12	0.05	0.22	0.04
3ONG	0.13	0.06	0.21	0.08
3ONL	0.19	0.11	0.16	0.11
3OUN	0.14	0.05	0.20	0.13
3P11	0.15	0.09	0.11	0.09
3PNL	0.11	0.03	0.14	0.08
3PRO	0.12	0.05	0.18	0.03
3PRP	0.19	0.05	0.21	0.07
3PRX	0.12	0.08	0.20	0.09
3PV6	0.14	0.01	0.20	0.03
3QC8	0.16	0.03	0.21	0.08
3QLU	0.13	0.03	0.15	0.03
3R66	0.15	0.04	0.16	0.02
3R9A	0.10	0.06	0.18	0.04
3RG6	0.11	0.04	0.19	0.06
3RJ3	0.13	0.03	0.15	0.06
3RNK	0.20	0.04	0.18	0.07
3RRM	0.13	0.10	0.14	0.08
3RVD	0.10	0.06	1.00	0.21
3S36	0.09	0.01	0.17	0.07
3S3X	0.13	0.04	0.28	0.06
3S5L	0.12	0.01	0.16	0.07
3SJV	0.15	0.04	0.17	0.08
3SM5	0.13	0.05	0.09	0.08
3T1Q	0.14	0.02	0.15	0.07
3T5G	0.12	0.06	0.16	0.04
3TG1	0.13	0.08	0.17	0.09
3UAI	0.14	0.03	0.18	0.06
3ULQ	0.14	0.08	0.23	0.05
3ULV	0.15	0.06	0.11	0.07
3VLB	0.12	0.04	0.11	0.04
3VU3	0.10	0.04	0.14	0.03
3VXU	0.15	0.05	0.16	0.05
3W31	0.18	0.06	0.15	0.05
3WIN	0.16	0.08	0.18	0.02
3WKT	0.16	0.03	0.16	0.07
3WWK	0.15	0.07	0.18	0.08
3WWN	0.12	0.02	0.41	0.12

3ZN6	0.11	0.04	0.13	0.04
3ZNZ	0.14	0.06	0.20	0.07
4BBN	0.14	0.04	0.19	0.11
4BI8	0.09	0.06	0.16	0.07
4BMP	0.15	0.03	0.18	0.07
4BOS	0.13	0.08	0.19	0.07
4BOZ	0.16	0.02	0.19	0.05
4BWS	0.13	0.09	0.21	0.06
4C0O	0.14	0.08	0.31	0.12
4C6T	0.14	0.06	0.19	0.07
4CPA	0.12	0.03	0.23	0.06
4CT4	0.14	0.04	0.14	0.05
4CU4	0.11	0.02	1.00	0.01
4DI3	0.14	0.05	0.14	0.02
4DN4	0.09	0.04	0.18	0.08
4DSS	0.11	0.04	0.14	0.03
4E2I	0.13	0.03	0.24	0.06
4EJX	0.14	0.04	0.13	0.07
4EMJ	0.12	0.05	0.17	0.06
4ETQ	0.09	0.05	0.13	0.06
4ETW	0.11	0.08	0.22	0.10
4EXT	0.16	0.02	0.19	0.04
4F7G	0.13	0.03	0.19	0.04
4FA8	0.10	0.05	0.18	0.09
4FI3	0.11	0.04	0.18	0.07
4FME	0.17	0.05	0.16	0.03
4FQR	0.11	0.03	0.10	0.05
4GAM	0.12	0.05	0.37	0.09
4GED	0.13	0.05	0.14	0.05
4GMJ	0.15	0.06	0.15	0.01
4GOJ	0.16	0.03	0.16	0.03
4GXU	0.12	0.03	0.09	0.05
4H8W	0.08	0.05	0.13	0.06
4HCN	0.16	0.04	0.27	0.07
4HH3	0.14	0.06	0.12	0.06
4HMY	0.15	0.04	0.19	0.02
4HQP	0.13	0.05	0.17	0.05
4HRE	0.14	0.06	0.16	0.05
4HX3	0.13	0.05	0.20	0.06
4I5L	0.12	0.05	0.14	0.04
4ICG	0.23	0.09	0.24	0.04
4IHF	0.11	0.02	0.23	0.07
4ILG	0.13	0.06	0.15	0.04
4ILH	0.13	0.02	0.12	0.09

4J38	0.15	0.05	0.13	0.04
4J4L	0.16	0.05	0.18	0.08
4JAV	0.16	0.05	0.16	0.02
4JCV	0.13	0.05	0.16	0.04
4JHP	0.12	0.09	0.16	0.05
4JO9	0.35	0.04	0.47	0.05
4JQW	0.18	0.07	0.19	0.06
4JX1	0.13	0.05	0.17	0.03
4K0V	0.16	0.05	0.14	0.03
4K71	0.15	0.05	0.15	0.06
4KEH	0.11	0.05	0.25	0.10
4KFM	0.11	0.04	0.14	0.02
4KI1	0.14	0.07	0.18	0.12
4KR0	0.11	0.05	0.12	0.07
4KXZ	0.10	0.03	0.13	0.03
4KYI	0.15	0.07	0.15	0.06
4L41	0.14	0.04	0.19	0.03
4LHU	0.13	0.05	0.15	0.07
4LLO	0.14	0.01	0.14	0.05
4LNU	0.12	0.05	0.15	0.04
4LRZ	0.14	0.05	0.15	0.07
4LSX	0.11	0.04	0.15	0.04
4LW4	0.10	0.04	0.16	0.08
4LX0	0.13	0.07	0.13	0.03
4M3K	0.13	0.05	0.14	0.02
4M5Z	0.08	0.05	0.13	0.04
4M7L	0.10	0.01	0.15	0.05
4MDK	0.13	0.04	0.19	0.07
4ML7	0.18	0.07	0.17	0.04
4MNE	0.13	0.04	0.12	0.06
4MRT	0.15	0.04	0.18	0.04
4N6O	0.14	0.03	0.18	0.07
4NBE	0.12	0.06	0.16	0.07
4NCO	0.13	0.03	0.08	0.04
4NIF	0.11	0.04	0.13	0.05
4NM8	0.11	0.08	0.11	0.02
4NYI	0.14	0.08	0.19	0.09
4NZL	0.17	0.02	0.17	0.05
4O4B	0.12	0.05	0.12	0.01
4O5I	0.13	0.04	0.09	0.04
4OL0	0.15	0.07	0.20	0.05
4P1B	0.10	0.04	0.15	0.10
4P2A	0.15	0.03	0.16	0.09
4P4H	0.15	0.06	0.17	0.08

4P4Q	0.12	0.06	0.14	0.05
4P69	0.14	0.05	0.16	0.06
4PDC	0.12	0.04	0.17	0.06
4PJ2	0.13	0.04	0.15	0.04
4PJ8	0.14	0.06	0.14	0.10
4POU	0.14	0.06	0.11	0.07
4PW9	0.12	0.02	0.21	0.05
4QD2	0.16	0.05	0.17	0.06
4QTI	0.10	0.03	0.18	0.10
4R4F	0.10	0.01	0.14	0.03
4R62	0.15	0.00	0.14	0.06
4R9Y	0.12	0.02	0.16	0.03
4RF0	0.14	0.04	0.25	0.08
4RIX	0.13	0.05	0.13	0.06
4RWS	0.11	0.05	0.20	0.07
4S10	0.16	0.06	0.20	0.10
4TXO	0.13	0.03	0.11	0.03
4TXV	0.13	0.03	0.15	0.03
4V2C	0.18	0.07	0.21	0.04
4WJG	0.12	0.04	0.18	0.07
4WLR	0.17	0.05	0.24	0.09
4WM0	0.13	0.02	0.44	0.02
4WW2	0.13	0.06	0.12	0.05
4WZA	0.08	0.04	0.14	0.05
4X6Q	0.15	0.04	0.16	0.05
4XHU	0.11	0.05	0.17	0.08
4XIG	0.12	0.05	0.13	0.04
4XKL	0.21	0.03	0.22	0.08
4XOI	0.13	0.04	0.18	0.05
4XR8	0.13	0.07	0.15	0.06
4XS0	0.14	0.03	0.10	0.05
4Y5O	0.23	0.09	0.28	0.09
4Y7M	0.13	0.03	0.11	0.01
4YEB	0.18	0.07	0.14	0.08
4YOC	0.13	0.08	0.16	0.08
4ZFR	0.15	0.02	0.24	0.08
4ZYP	0.11	0.04	0.11	0.06
5AUP	0.13	0.05	0.24	0.07
5BMU	0.15	0.06	0.16	0.06
5BRR	0.13	0.06	0.17	0.07
5BVP	0.10	0.02	0.16	0.10
5BXQ	0.15	0.02	0.10	0.03
5C3I	0.18	0.06	0.20	0.07
5C7X	0.10	0.02	0.19	0.09

5CEC	0.10	0.02	0.13	0.07
5CNV	0.12	0.03	0.13	0.05
5CRA	0.16	0.04	0.21	0.06
5CVO	0.15	0.04	0.15	0.09
5E0K	0.10	0.07	0.12	0.08
5E8D	0.11	0.07	0.20	0.12
5EE4	0.11	0.04	0.10	0.04
5EG3	0.14	0.03	0.21	0.04
